# How do the Hückel and Baird Rules Fade away in Annulenes?

**DOI:** 10.3390/molecules25030711

**Published:** 2020-02-07

**Authors:** Irene Casademont-Reig, Eloy Ramos-Cordoba, Miquel Torrent-Sucarrat, Eduard Matito

**Affiliations:** 1 Donostia International Physics Center (DIPC), 20018 Donostia, Euskadi, Spain; irenecasre@gmail.com (I.C.-R.); eloy.raco@gmail.com (E.R.-C.); miqueltorrentsucarrat@gmail.com (M.T.-S.); 2 Kimika Fakultatea, Euskal Herriko Unibertsitatea (UPV/EHU), 20080 Donostia, Euskadi, Spain; 3 IKERBASQUE, Basque Foundation for Science, 48013 Bilbao, Euskadi, Spain

**Keywords:** annulenes, aromaticity, antiaromaticity, Hückel rule, Baird rule, density functional theory, delocalization error

## Abstract

Two of the most popular rules to characterize the aromaticity of molecules are those due to Hückel and Baird, which govern the aromaticity of singlet and triplet states. In this work, we study how these rules fade away as the ring structure increases and an optimal overlap between *p* orbitals is no longer possible due to geometrical restrictions. To this end, we study the lowest-lying singlet and triplet states of neutral annulenes with an even number of carbon atoms between four and eighteen. First of all, we analyze these rules from the Hückel molecular orbital method and, afterwards, we perform a geometry optimization of the annulenes with several density functional approximations in order to analyze the effect that the distortions from planarity produce on the aromaticity of annulenes. Finally, we analyze the performance of three density functional approximations that employ different percentages of Hartree-Fock exchange (B3LYP, CAM-B3LYP and M06-2X) and Hartree-Fock. Our results reveal that functionals with a low percentage of Hartree-Fock exchange at long ranges suffer from severe delocalization errors that result in wrong geometrical structures and the overestimation of the aromatic character of annulenes.

## 1. Introduction

Aromaticity is one of the most important concepts in chemistry, and it is associated with cyclic electron delocalization (or conjugation) in closed circuits giving rise to bond-length equalization, energy stabilization, large magnetic anisotropies and abnormal chemical shifts, among other well-known effects [1,2,3]. Despite the multidimensional character of aromaticity and the recent proliferation of aromatic compounds that extend beyond the organic chemistry realm [4,5], several simple but predictive models have been designed to characterize aromaticity [6,7,8,9,10]. From these models, *rules of aromaticity* [11] have been obtained that are commonly used in assessing the aromatic character of molecules. Among these rules, the Hückel rule was the first and remains the most widely employed [12,13,14,15].

According to the Hückel rule, annulenes with 4n+2 π electrons (*n* being an integer number) are more stable than the corresponding open-chain polyenes and, therefore, considered *aromatic*. Although this prediction is met for the first elements of the annulenes series, the rule is soon broken due to out-of-plane geometrical distortions that are more energetically favorable but disrupt the optimal overlaps between *p* orbitals that give rise to conjugated circuits [16]. These geometrical distortions cannot be considered by the Hückel rule which, inevitably, overestimates the aromaticity of 4n+2 annulenes that are described as planar structures having bond equalization. Conversely, the Hückel rule also predicts that 4n π electrons annulenes are unstable with respect to their open-chain analogues, exhibit bond length alternation, and are considered *antiaromatic*. The application of this rule has not been limited to annulenes and it is currently used in all sorts of molecules, including non-organic molecules [4,5].

Another important rule of aromaticity, complementary to Hückel’s, is the Baird rule [17]. While the Hückel rule is limited to singlet ground states, the Baird rule applies to the lowest-lying triplet and excited singlet states and predicts 4n (4n+2) π-electron molecules to be aromatic (antiaromatic). Aihara calculated the resonance energies of singlet and triplet annulenes, concluding that Baird’s rule was satisfied, i.e., that the lowest-lying excited state of annulenes presented the opposite aromatic character to that in the ground state [18]. Baird’s rule has been called the *photochemistry analogue of Hückel’s rule* [19] because it has been used to tailor molecules with enhanced photochemical activity [20,21,22].

The most (anti)aromatic molecules do not usually have many atoms in the ring. The optimal overlap between orbitals that favors electron conjugation calls for a particular arrangement of the atoms in the ring that cannot be sustained as the ring size increases. Therefore, both aromaticity and antiaromaticity are expected to decrease with the ring size. Although there is evidence of large aromatic nanorings [23], studies on annulenes have led to the conclusion that systems with more than 30π electrons are nonaromatic [24].

In his seminal paper, Baird studied the velocity at which the antiaromaticity (measured through Dewar resonance energy, DRE) diminished with increasing ring size [17]. Based on a few examples, he found that 4n+2 triplets lost their antiaromaticity with the ring size as fast as 4n singlets [17]. To the best of our knowledge, thus far, there have been very few attempts to actually quantify how the aromaticity rules fade away with the ring size and they were limited to 4n+2 annulenes [25,26]. This is the goal of this paper. We will first perform an analysis of how these rules are expected to change with the ring size as predicted by the model from which they originated. Second, we will study how the geometry distorts these molecules from the optimal (planar) geometry assumed in the model and how it affects the aromaticity. Finally, we will analyze the effect of the computational method in the description of these systems by taking into account several density functional approximations (DFAs) with a varying percentage of Hartree Fock (HF) exchange.

## 2. Methodology

### 2.1. Aromaticity Indices

In this section, we will review the expressions of several aromaticity indices based on electron delocalization [2]. In the following, we will assume a molecule having at least one ring structure, which consists of *N* atoms represented by the string A = {$A_{1}$,$A_{2}$,...,$A_{N}$}, whose elements are ordered according to the connectivity of the atoms in the ring. For convenience, we adopt $A_{N+p}\equiv A_{p}$ and $A_{0}\equiv A_{N}$.

#### 2.1.1. The Aromatic Fluctuation Index: FLU

The FLU index measures aromaticity by comparison with the cyclic electron delocalization of some reference aromatic molecules [27]. Its expression depends on the delocalization index (DI) [28,29,30,31], δ(A,B), which measures the electron sharing between atoms *A* and *B*,

(1) $$\begin{array}{c} FLU\left(A\right)=\frac{1}{N}\sum_{i=1}^{N}{\left[{\left(\frac{\delta (A_{i})}{\delta (A_{i-1})}\right)}^{\alpha }\left(\frac{\delta \left(A_{i},A_{i-1}\right)-\delta_{ref}\left(A_{i},A_{i-1}\right)}{\delta_{ref}\left(A_{i},A_{i-1}\right)}\right)\right]}^{2}\;, \end{array}$$

where δ(A) is the atomic valence for a closed-shell system [32], and α is a simple function to ensure that the first term in Equation (1) is always greater or equal to 1,

(2) α=1δ(Ai-1)≤δ(Ai)-1δ(Ai)<δ(Ai-1)

$\delta_{ref}\left(A,B\right)$ is the DI corresponding to an aromatic molecule which has the pattern of bonding A-B. For example, the aromatic reference for C-C bonds is benzene. FLU is close to zero for aromatic species, and greater than zero for non-aromatic or antiaromatic species.

Aromaticity indices based on references, such as FLU or HOMA [33,34], do not measure aromaticity but the similarity with respect to some aromatic molecule. Therefore, they are not adequate to describe reactivity [35,36].

#### 2.1.2. The Bond-Length and Bond-Order Alternation Indices

Two popular indicators of aromaticity are the bond-length (BLA) and the bond-order alternation (BOA), which compare the average of bond lengths and bond orders, respectively, of consecutive bonds in a ring:

(3) $$\frac{1}{n_{1}}\sum_{i=1}^{n_{1}}x_{A_{2i-1},A_{2i}}-\frac{1}{n_{2}}\sum_{i=1}^{n_{2}}x_{A_{2i},A_{2i+1}},$$

where $n_{1}=\left\lfloor \left(N+1\right)/2\right\rfloor$ and $n_{2}=\left\lfloor N/2\right\rfloor$, ⌊y⌋ being the floor function of *y*, that returns the largest integer less than or equal to *y*, and *x* is either the bond length or the bond order. Unlike the atomic charges [37], the bond orders are much less dependent on the computational method and the basis set used [38] and, therefore, aromaticity indices based on bond orders [39] are not highly dependent on the level of theory employed. An exception to this rule is the delocalization error that is present in some DFAs [40] with a low percentage of Hartree-Fock exchange, which causes an overestimation of the aromaticity of some compounds (see Section 3.4).

The definition of Equation (3) presents a serious drawback: it is not well defined for a ring of an odd number of members because its value depends on the order of the atoms in the ring. For the sake of generality, in this paper we adopt an alternative definition of BLA/BOA:

(4) $$\frac{1}{2N}\sum_{i=1}^{N}\left|x_{A_{i},A_{i+1}}-x_{A_{i+1},A_{i+2}}\right|.$$

and we choose the delocalization index [28,29,30,31] as a measure of bond order.

#### 2.1.3. A Many-Center Electron Delocalization Index: $I_{\mathrm{ring}}$ 

Giambiagi and coworkers suggested to use the multicenter index, which was previously defined by them to account for the simultaneous electron sharing among various centers [41], as a measure of aromaticity [42]. This index was named $I_{\mathrm{ring}}$ and its formulation for single-determinant wavefunctions reads as follows:

(5) $$\begin{array}{c} I_{\mathrm{ring}}\left(A\right)=\sum_{i_{1},i_{2},\ldots ,i_{n}}^{occ}S_{i_{1}i_{2}}\left(A_{1}\right)S_{i_{2}i_{3}}\left(A_{2}\right)\ldots S_{i_{n}i_{1}}\left(A_{n}\right), \end{array}$$

where $S_{ij}\left(A\right)$ is the overlap of molecular orbitals *i* and *j* in the atom *A*, i.e.,

(6) $$\begin{array}{c} S_{ij}\left(A\right)=\int_{A}\phi_{i}^{*}\left(r\right)\phi_{j}\left(r\right)dr. \end{array}$$

$I_{\mathrm{ring}}$ will provide large values for aromatic molecules. Although it will not be considered in this paper, it is worth to mention that Bultinck and co-workers generalized $I_{\mathrm{ring}}$ considering also the delocalization of a non-Kekule arrangement of the atoms in the ring; the index is known as MCI [43]. Some of us have shown that both $I_{\mathrm{ring}}$ and MCI are ring-size dependent [44] and, therefore, for convenience, we will calculate the multicenter *electron delocalization per atom* that can be obtained as $I_{\mathrm{ring}}$ ${}^{1/N}$.

#### 2.1.4. AV1245 and ${\mathrm{AV}}_{\min}$ 

Both $I_{\mathrm{ring}}$ and MCI are among the less fallible aromaticity indices available in the literature [2,36,45] and, therefore, they have been used in a plethora of cases involving a difficult assessment of aromaticity [46,47,48,49,50,51,52,53,54]. However, these indices present some drawbacks that prevent their use in large rings [55] and, therefore, we have recently designed [55] and tested [40,56] a new electronic aromaticity index, AV1245, based on MCI but free of the shortcomings of this index. AV1245 is defined as the average value of the four-atom MCI index between relative positions 1–2 and 4–5 constructed from each five-atom fragment (five consecutive atoms) along the perimeter of the ring [55]. The latter gives an *average picture* of the electron delocalization among the atoms in the ring. However, for the purpose of measuring aromaticity, it has been found more adequate to use the minimum absolute MCI value evaluated along the perimeter, ${\mathrm{AV}}_{\min}$ [56]. ${\mathrm{AV}}_{\min}$ identifies the *weakest link*, i.e., the fragment with the lowest electron delocalization which is usually responsible for the loss of aromaticity in the ring. A detailed study of all the MCI values along the perimeter has been recently shown to be useful in identifying electron delocalization patterns and it will be the topic of discussion in a forthcoming work. Aromatic molecules are thus identified by large values of ${\mathrm{AV}}_{\min}$ (${\mathrm{AV}}_{\min}$ ≫1) and non-aromatic molecules exhibit very low ${\mathrm{AV}}_{\min}$ values. Antiaromatic molecules usually exhibit intermediate ${\mathrm{AV}}_{\min}$ values and are more difficult to identify. To this end, the analysis is complemented by the examination of either four-atom MCI profiles along the perimeter of the ring or the BOA, which help differentiate between aromatic and antiaromatic compounds.

### 2.2. Hückel Molecular Orbital Method

Despite the drastic approximations inherent in the Hückel Molecular Orbital (HMO) approach [12,13,14,15], organic aromatic molecules are usually well described within the HMO method. It is thus usual to learn the HMO method at the same time as 4n+2 Hückel’s rule and other aromaticity measures given by the HMO method, such as the resonance energy (RE), the RE per electron (REPE) or the topological REPE (TREPE) [57]. Some recent works have studied Hückel’s rule from the perspective of electron delocalization [58,59]. Since the studies of Hückel on organic molecules, the concept of aromaticity has extended importantly including all sorts of new aromatic molecules such as metalloaromatic molecules [4,51,52,60,61], fullerens [62], nanotubes [63], porphyrins [64,65] with a Möbius-like structure [66,67,68,69,70,71,72,73], and all-metal clusters [4,5], among others.

For a cyclic polyene of *n* carbon atoms and *N* π electrons, the Hückel molecular orbital (HMO) method provides a general formulation for its orbitals:

(7) $$\phi_{l}=\sum_{\mu =1}^{N}\chi_{\mu }c_{\mu l}=\frac{1}{\sqrt{N}}\sum_{\mu =1}^{N}\chi_{\mu }e^{2\pi i(\mu -1)l/N},$$

where $\chi_{\mu }$ is the atomic orbital 2$p_{z}$ of the $\mu^{\mathrm{th}}$ carbon atom and l=0±1,…,±(N-1)/2,N/2. Excepting for the first ($\phi_{0}$) and the last ($\phi_{N/2}$), these orbitals are complex and degenerate by pairs ($\phi_{\pm 1},\phi_{\pm 2},\ldots$). From these equations, one can easily calculate the atomic overlap matrices (AOMs), the bond orders, as well as many aromaticity indices (see Section 2.1), obtaining analytical expressions [74].

The typical way to assess the aromaticity within the framework of HMO theory is through the study of stabilization or resonance energy. Since the computational chemistry study of resonance energies puts severe limitations to analyze more complicated molecules, especially those containing atoms other than carbon, we prefer to study electron delocalization to assess the aromatic character of compounds [2,55]. In particular, we focus here on the study of $I_{\mathrm{ring}}$ which, in the case of HMO theory, takes a very simple form (compare to Equation (5)),

(8) $$\begin{array}{c} I_{\mathrm{ring}}^{\mathrm{HMO}}\left(A\right)=P_{A_{1}A_{2}}P_{A_{2}A_{3}}\ldots P_{A_{n}A_{1}}, \end{array}$$

where $P_{AB}$ is the bond-order between atoms *A* and *B* and it is related to the DI: $\delta \left(A,B\right)=P_{AB}P_{BA}$ [74].

In order to compare annulenes of different sizes among them, we will study the normalized quantity $I_{\mathrm{ring}}$ ${}^{1/N}$ [44]. The calculation of the bond orders takes a very simple form for singlet annulenes with 4n+2π electrons [74]. The study of other multiplicities or number of π electrons is less evident. The HMO theory does not distinguish between alpha and beta electrons (beyond the fact of permitting only one electron of each kind to populate each orbital) and, therefore, the study of triplets is not entirely satisfactory. However, to a reasonable extent, one can analyze the 4n+2 triplets as cases where one electron is promoted from a *l* orbital to a l+1 orbital. If the orbitals obtained from HMO are those of Equation (7), the results are the same regardless the electrons are promoted from *l* or -l orbitals (or whether they are promoted to l+1 or -l-1 orbitals). Hence, the study of 4n+2 triplets can be done without ambiguity. However, the study of neutral 4n singlets, which are expected to exhibit localized structures of symmetry $D_{\frac{N}{2}h}$, cannot be conducted because both *l* and -l orbitals contribute the same amount to all the bond orders in the perimeter of the ring. In order to enforce the appearance of mesomeric structures, we should obtain a different set of orbitals from the HMO theory. Since degenerate orbitals can be freely combined without altering the energy of the system, it is convenient to recombine each pair of degenerate orbitals among themselves to produce this set of orbitals:

(9) $${\bar{\phi }}_{l}=\left(1+i\right)\phi_{l}+\left(1-i\right)\phi_{-l},$$

for l=±1,…,±(N-1)/2. Unlike those in Equation (7), these orbitals are real and produce one of the two mesomeric structures that one can expect in neutral 4n annulenes, depending on whether the last two electrons occupy a *l* or -l orbital. Finally, we find that the study of neutral 4n triplets within the HMO theory is far from obvious because both the singlet-open and the triplet states would be actually described by the same orbital occupancies. For the sake of completeness, we have also included this case in our study. To this end, we have employed the original set of orbitals (Equation (7)) and these results are labeled as 4n $D_{Nh}$. (We could have also chosen the second set of orbitals that lead to mesomeric structures. In such case, we would have obtained different $I_{\mathrm{ring}}$ values but the same qualitative trend (see Section 3.1)).

## 3. Results

### 3.1. Aromaticity from the HMO Method

First of all, we will study Hückel’s and Baird’s rules from the simple HMO theory [12,14]. Although we cannot expect this theory to provide a reliable description of annulenes (especially the large ones), the results we obtain will provide a high bound to the aromaticity/antiaromaticity expected in these species.

**Figure 1:**
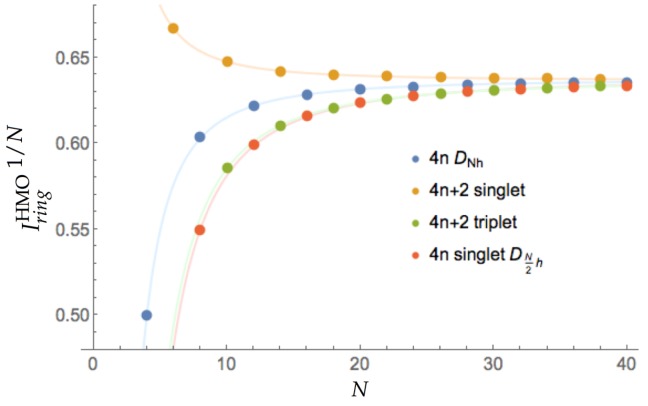
Values of $I_{\mathrm{ring}}$${}^{1/N}$ for the annulenes series against the number of C atoms (*N*) for different singlets and triplets. The species have been divided according to the number of electrons (4*n* and 4*n* + 2) and the spin multiplicity (singlet and triplet).

All the values of $I_{\mathrm{ring}}$ (Equation (8)) are collected in Figure 1. Interestingly, all the curves conform to the same general expression: $a+b/N^{2}$, where *N* is the number of ring members. The values of *a* do not differ significantly among the four cases giving values very close to the theoretical limit for annulenes, 2/π, whereas the values of *b* present large differences among them: −2.1867, 1.0822, −5.1151, and −5.5910 for 4n $D_{Nh}$, 4n+2 singlet, 4n+2 triplet, and 4n singlet $D_{\frac{N}{2}h}$ structures, respectively. These results are in agreement with the chemical intuition: (*i*) for very large annulenes both Hückel and Baird rules break and all the species become equally aromatic, (ii) the initial (anti)aromatic character decreases smoothly with the annulene size. We can also see that 4n+2 singlets, which are aromatic, display values above the 2/π limit, whereas 4n+2 triplets and 4n singlets, which are expected to be antiaromatic, present values below this limit. On the other hand, 4n$D_{Nh}$ annulenes also behave like antiaromatic molecules, which does not conform with the aromaticity expected in 4n triplets and, therefore, these species are not well described from the delocalization measures we can obtain from the HMO theory. Finally, we can compare the velocity with which the (anti)aromaticity decreases according to Hückel’s and Baird’s rule. The values of *b* show that the velocity at which the aromaticity decreases with the ring size is about five times smaller for 4n+2 singlet annulenes than the corresponding decrease of antiaromaticity for 4n+2 triplets. Interestingly, the latter is very close to the decrease of antiaromaticity found in 4n singlets, in agreement with the study of Baird on cyclic hydrocarbons using DRE [17]. We have also studied the HMO method forcing bond-length alternation by employing two different resonance integrals for each bond type (single and double) [75,76], finding that the antiaromaticity of 4n singlets decreases more rapidly when the annulenes are forced to exhibit bond alternation. Since we cannot compare 4n singlets and triplets, we cannot extract any relevant conclusion about which rule, Hückel’s or Baird’s, is broken more quickly with the ring size. We can, however, conclude from this data that molecules are more resilient to the loss of aromaticity than to the loss of antiaromaticity, as one would expect from purely energetic grounds.

### 3.2. Geometrical Relaxation

**Figure 2:**
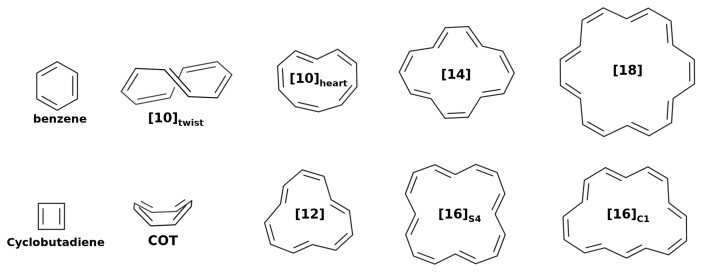
Geometrical structures of the studied annulenes.

Thus far, we have studied ideally planar annulenes within the HMO method. In this section, we employ quantum chemistry methods to study the geometry of annulenes, which often do not attain a planar conformation in their ground state configuration [77] (see Figure 2). We will employ HF and three DFAs: B3LYP, M06-2X and CAM-B3LYP. These results will be compared against the benchmark data available in the literature. We will split the results into two blocks: 4n+2 and 4nπ-electron annulenes.

#### 3.2.1. 4n+2 Annulenes

First of all, we have studied the lowest-lying structures of singlet and triplet benzene. The ground state of benzene has been largely studied and its characterization does not present a challenge for DFAs. The molecule is identified by all the aromaticity measures as the most aromatic molecule among the studied annulenes. Conversely, the triplet state of benzene has a more elusive character, presenting two $D_{2h}$ conformations which are very close in energy, the quinoidal (Q) and antiquinodial (AQ) one [17]. The former is characterized by two clear double bounds separated by two radical C atoms, whereas the AQ shows two allylic structures merged by two single C-C bonds. The energy difference between the two conformations is below 1 kcal/mol and they are connected by a transition state with an energy barrier of 2.5 kcal/mol [78]. Both conformers are expected to be antiaromatic, displaying an energy destabilization that is responsible for their prominent photochemical reactivity [79]. The lowest-lying triplet state of benzene has been identified by HF, B3LYP, and CAM-B3LYP as AQ-like, whereas the Q structure could not be located with these methods. Conversely, M06-2X provides a Q-like structure. The aromaticity indices display values which are much smaller than those of benzene and the BOA index reveals a bond-order alternation that is characteristic of antiaromatic molecules. ${\mathrm{AV}}_{\min}$ values are small, which in conjunction with the oscillating pattern displayed by the dissected index profile (see Supplementary Material), agrees with the antiaromatic character anticipated in this species.

Singlet [10]annulene presents several low-energy isomers, which are difficult to sort energetically from the results of computational calculations. Most ab initio methods find the *twist* conformation to be the lowest in energy, the exception being B3LYP, which predicts the *heart* conformation to be the ground state [16]. Our calculations agree with these results. The most delocalized structure, corresponding to a $D_{10h}$ symmetry, lies more than 30kcal/mol above the ground state geometry [16] and, hence, the isomer expected to be the most aromatic it is actually not stable. On the contrary, the twist isomer (which does not exhibit a planar conformation) presents a very modest aromatic/antiaromatic character according to all the aromaticity indices. In fact, the molecule could be easily classified as nonaromatic from the ${\mathrm{AV}}_{\min}$ value, which is very close to zero and, as the dissected index profile shows (see Supplementary Material), the twisted bonds are those that prevent an optimal overlap of *p* orbitals. Conversely, the heart configuration (lying 0.6 and 2.3 kcal/mol above the ground state according to CAM-B3LYP and M06-2X) is an aromatic molecule as stated by all aromaticity indices. To the best of our knowledge, triplet [10]annulene has been much less studied and there are only examples of triplet dicationic [10]annulene structures on the literature [20,21]. The lowest-lying triplet state of [10]annulene displays *naphthalene* (B3LYP, CAM-B3LYP and M06-2X) and *twist* (HF) configurations. Following the Baird rule, this molecule should be antiaromatic. The naphthalene conformation is indeed mildly antiaromatic but the twist conformation is rather nonaromatic (see Table 1).

The ground-state [14]annulene geometry corresponds to a distorted pyrene perimeter [16] that can undergo a facile isomerization reaction through a Möbius antiraromatic transition state [80]. At the B3LYP level of theory, we locate a minimal structure with $C_{2h}$ symmetry that is aromatic according to all criteria of aromaticity. However, the CAM-B3LYP and M06-2X $C_{2h}$ configurations correspond to a transition state (TS) that through a small energy barrier (ca. 2.5 kcal/mol) connects two $C_{s}$-symmetry antiaromatic energy minima with complementary bond-length alternation. The triplet conformer leads to an energy minimum that is only mildly antiaromatic at all the levels of theory.

The Hückel rule predicts that [18]annulene is an aromatic molecule but the situation is similar to [14]annulene. B3LYP finds the molecule to be a highly symmetric ($D_{6h}$) structure that is also aromatic from all aromaticity indices. However, both CAM-B3LYP and M06-2X predict that this structure is actually a transition state that connects two identical structures that exhibit bond-order alternation and, therefore, are antiaromatic. This discrepancy has been attributed to the delocalization error of DFAs with a low percentage of Hartree-Fock exchange, a fact that has been confirmed through the comparison of experimental proton chemical shifts [81]. A detailed study of the delocalization error is done in Section 3.4 of this manuscript. The lowest-lying triplet displays a $D_{2}$ symmetry (HF) or a $C_{2h}$ symmetry (CAM-B3LYP, B3LYP, and M06-2X). DFAs agree on the triplet [18]annulene being weakly antiaromatic, whereas HF predicts it to be rather nonaromatic.

#### 3.2.2. 4n Annulenes

The photochemical formation of 4n annulenes is very important in excited-state aromaticity [19]. Cyclobutadiene is often used as the paradigmatic example of an antiaromatic molecule following the 4n rule. Although (anti)aromaticity can be easily overestimated with a single-determinant wavefunction [82,83,84], cyclobutadiene is the molecule with the largest bond-length and bond-order alternation and all indicators of aromaticity clearly confirm its antiaromatic character. Baird was first to suggest that triplet 4n π-electron annulenes should be regarded as aromatic and confirm it through DRE calculations [17]. In particular, the DRE confirmed that triplet cyclobutadiene is an aromatic molecule. A fact that is further substantiated by its symmetric $D_{4h}$ geometry and the values of the aromaticity indices gathered in Table 2.

Unlike cyclobutadiene, cycloocta-1,3,5,7-tetraene (COT) is not a planar molecule in its ground state. COT shows a boat-like $D_{2d}$ geometry that, although presents bond-length and bond-order alternation, is not a very antiaromatic molecule [16,85]. The aromaticity indices reveal that this molecule could be classified as mildly antiaromatic. Conversely, the lowest-lying triplet state of COT presents $D_{8h}$ symmetry and values of the aromaticity indices that confirm the aromatic character anticipated by the Baird rule. Interestingly, the realization of planar triplet COT in some substituted annulenes has been studied as an acceleration path for the photochemical inversion of the ring [22].

In contrast to smaller annulenes, [12]annulene presents several energy minima in the potential energy surface, five of which lie within 5 kcal/mol according to CCSD(T) calculations [86]. The instability and the easy isomerization [87] of this species explain the difficulty in characterizing it experimentally. All DFAs and HF predict a CTCTCT ($C_{1}$) geometry with a large bond-order alternation, where C and T stand for the arrangement of C-C bonds that can be either cis (C) or trans (T). Nevertheless, the molecule presents very small ${\mathrm{AV}}_{\min}$ values that indicate a nonaromatic character. The lowest-lying triplet state of [12]annulene shows a very similar geometry in agreement with a CTCTCT arrangement of the atoms but corresponding to a CTCTCT ($C_{s}$) geometry, according to all DFAs. The HF lowest-lying triplet also presents a CTCTCT geometry ($C_{1}$) but it is severely distorted with respect to the latter one. Regardless of the method used, ${\mathrm{AV}}_{\min}$ value is small, prompting us to also classify the triplet state as a nonaromatic species.

As in the previous case, a large number of [16]annulene isomers have been found [88] and, therefore, this species also easily undergoes isomerization [87] even through quantum mechanical tunneling [89]. According to all the methods, the ground state structure can be classified as CTCTCTCT ($S_{4}$). Interestingly, there is another structure, which presents a distorted CTCTTCTT structure ($C_{1}$) lying 5-8 kcal/mol above the ground state structure. The lowest-lying triplet presents a conformation very close to the latter one. Although the singlet is less antiaromatic than COT or cyclobutadiene, we find that both $S_{4}$ and $C_{1}$ present a similar character and are more antiaromatic than [12]annulene. Therefore, [16]annulene could be classified as weakly antiaromatic. Likewise, the triplet provides values of the indices that indicate a more aromatic character than [12]annulene triplet. Although ${\mathrm{AV}}_{\min}$ values oscillate between 0.35 and 1.16, these values indicate a mild aromatic character in between triplet [12]annulene and triplet cyclobutadiene, which agrees with the fact that this molecule displays a more symmetric structure ($C_{s}$) than its singlet counterpart ($C_{1}$).

### 3.3. Aromaticity from DFAs

**Figure 3:**
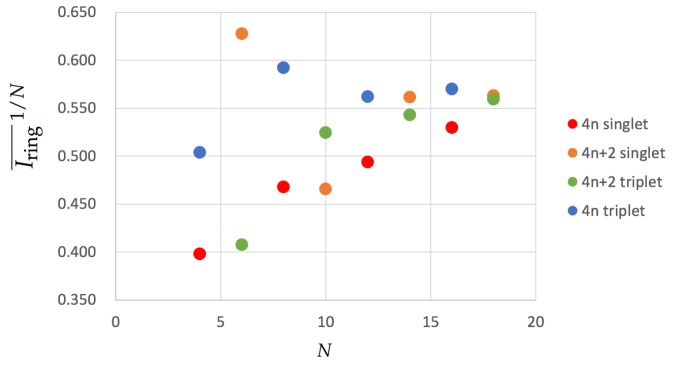
Values of $\bar{I_{\mathrm{ring}}}$ ${}^{1/N}$ for the annulenes series in terms of the number of C atoms (*N*) for the lowest-lying singlets and triplets. The species have been divided according to the number of electrons (4*n* and 4n+2) and the spin multiplicity (singlet and triplet). Calculations were performed with CAM-B3LYP/6-311G(d,p).

The HMO cannot take into account the geometrical relaxation from a planar structure (permitting an optimal overlap of *p* orbitals) and, therefore, the results obtained in the Section 3.1 are upper bounds to aromaticity and antiaromaticity in annulenes. In this section, we will consider the optimization of several singlet and triplet annulenes and how it affects the aromaticity of these compounds. As an improvement over HMO results, we will calculate an approximate version of $I_{\mathrm{ring}}$ that consists in using only the two-center bond orders of bonded atoms to estimate the *N*-center delocalization. In particular, we will employ the equivalent of Equation (8) in the framework of quantum mechanics. To this end, we will take δ(A,B) and subtract 1.00 to (approximately) remove the sigma contribution to the C-C bond, and multiply all resulting DIs of the bonds around the perimeter of the ring,

(10) $$\begin{array}{c} \bar{I_{\mathrm{ring}}}\left(A\right)=\sqrt{\left(\delta (A_{1},A_{2})-1\right)\left(\delta (A_{2},A_{3})-1\right)\ldots \left(\delta (A_{n},A_{1})-1\right)}, \end{array}$$

where we have taken the square root to account for the fact that at the HMO level the DI corresponds to the square of the Hückel bond-order [74]. One can consider the latter as a rough approximation that bridges the HMO definition (Equation (8)) and the actual $I_{\mathrm{ring}}$ value (Equation (5)). We have collected the results of $\bar{I_{\mathrm{ring}}}$ ${}^{1/N}$ in Figure 3.

The results show some differences with respect to the HMO values we have shown earlier. 4n+2 singlet compounds show a similar trend to the HMO results except for [10]annulene, which exhibits a smaller aromaticity than expected. This molecule undergoes important geometrical distortions that disrupt the overlap of *p* orbitals and causes the apparent loss of aromaticity. 4n singlets are expected to be antiaromatic, as is confirmed by the large BOA values (see Table 1 and Table 2), and they become less and less antiaromatic as they increase the ring size (see the increasing $\bar{I_{\mathrm{ring}}}$ ${}^{1/N}$ values of Figure 3). 4n+2 triplet annulenes follow a very similar trend in agreement with Baird’s rule. Finally, we examine the 4n triplet annulenes that are expected to be aromatic and for which there is no clear trend. On one hand, COT, which displays a planar structure, is actually among the most aromatic compounds, even more aromatic than cyclobutadiene in its lowest-lying triplet state, which actually shows the smallest value among the molecules that are expected to be aromatic. On the other hand, neither [12] nor [16]annulene present a planar structure and, therefore, they exhibit larger BOA values, suggesting that these molecules are rather nonaromatic or slightly antiaromatic. Interestingly, all the aromaticity trends are similar to those found for pure HMO calculations for 4n+2 annulenes and 4n singlets with the only mentioned exception of [10]annulene.

Table 1 and Table 2 also include the actual values of $I_{\mathrm{ring}}$ ${}^{1/N}$ but we have not discussed them because they mostly corroborate the trends we have found with $\bar{I_{\mathrm{ring}}}$ ${}^{1/N}$ and some of the values for large annulenes could not be obtained with enough precision. This is one of the main shortcomings of $I_{\mathrm{ring}}$ for large rings [55], the values become so small that they conflict with the numerical precision of the calculation and, consequently, we cannot obtain a reliable normalization ($I_{\mathrm{ring}}$ ${}^{1/N}$) which permits the comparison among rings of different sizes.

**Figure 4:**
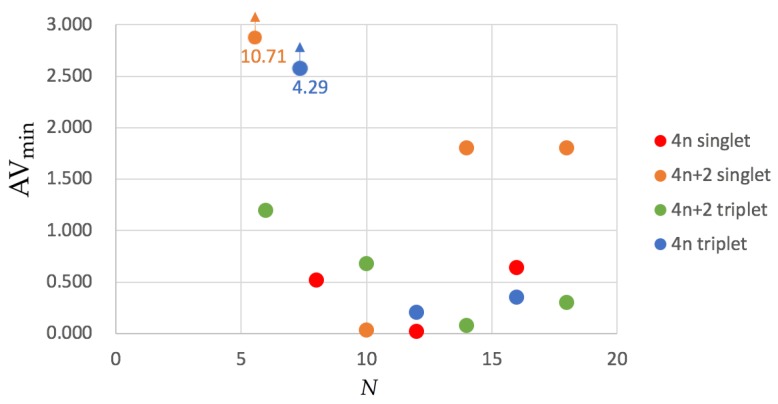
Values of ${\mathrm{AV}}_{\min}$ for the annulenes series (lowest-lying singlets and triplets) in terms of the number of C atoms (*N*). The species have been divided according to the number of electrons (4*n* and 4n+2) and the spin multiplicity (singlet and triplet). Calculations were performed with CAM-B3LYP/6-311G(d,p).

In order to remedy this problem, ${\mathrm{AV}}_{\min}$ was recently designed [55]. ${\mathrm{AV}}_{\min}$ values for the annulenes series are collected in Figure 4. Although the trends are not so clear as in HMO calculations or the approximate account of aromaticity with $\bar{I_{\mathrm{ring}}}$ ${}^{1/N}$, the picture we can extract from ${\mathrm{AV}}_{\min}$ does not differ entirely from that we obtained from $\bar{I_{\mathrm{ring}}}$ ${}^{1/N}$. The four most aromatic molecules are the same according to both indices and each single-triplet pair shows the same order of aromaticity, e.g., singlet benzene is more aromatic than triplet benzene. The only apparent exception to this rule is [10]annulene that has a larger ${\mathrm{AV}}_{\min}$ value for the lowest-lying triplet than for the ground state singlet. The latter is due to the fact that the ground state configuration of [10]annulene is found to be nonaromatic (the second lowest-lying structure, the *heart* configuration, is actually quite aromatic) whereas the triplet configuration is antiaromatic.

Finally, we find that FLU, BOA, and BLA give a similar qualitative trend to $\bar{I_{\mathrm{ring}}}$ ${}^{1/N}$ (see Table 1 and Table 2, and also the Supplementary Material) with the exception of large 4n+2 annulenes ([14]annulene and [18]annulene) that are found to be more aromatic in their triplet state than in their ground state. One should bear in mind that these indices are based on pairwise interactions (FLU is also based on fixed ground-state aromatic references) and they are not as reliable as indices based on multicenter calculations [36].

### 3.4. The Delocalization Error in DFAs

DFAs suffer the so-called delocalization error [90,91,92], which tends to give delocalized electronic structures. This is the exact opposite behavior of HF, which tends to overestimate the localization of the electronic structures. DFAs with a low percentage of HF exchange are more prone to delocalization errors. This effect has been observed in conjugate systems [93] and aromatic systems [40,81,94,95] including singlet annulenes [81]. Recently, Contreras-García, et al. [96] have suggested that the exact result is often bracketed between two extreme situations, the highly localized one (HF) and a highly delocalized one (LDA, which does not include HF exchange). They have used this result to construct error bars for the calculation of some solid-state properties. Likewise, Burke, et al. [97] have defined the sensitivity of DFAs to the density as the energy difference between DFA calculations from LDA and HF densities.

In this section, we investigate how the delocalization error affects the aromaticity measures in the studied annulene series through the analysis of $\bar{I_{\mathrm{ring}}}$ ${}^{1/N}$ calculated with four methods that use a different amount of HF exchange. In our study, LDA is ruled out because it is not reliable for gas-phase calculations [98] and B3LYP is used as the most *delocalizing* method (includes 19% of HF exchange). As the most *localizing* method we employ HF (that obviously uses 100% of HF exchange), and, as DFA with a large percentage of HF exchange, we have selected M06-2X (54%) and a range-separation functional [91], CAM-B3LYP, which presents a variable amount of HF exchange that goes from 19% at short ranges to 65% at large interelectronic separations.

**Figure 5:**
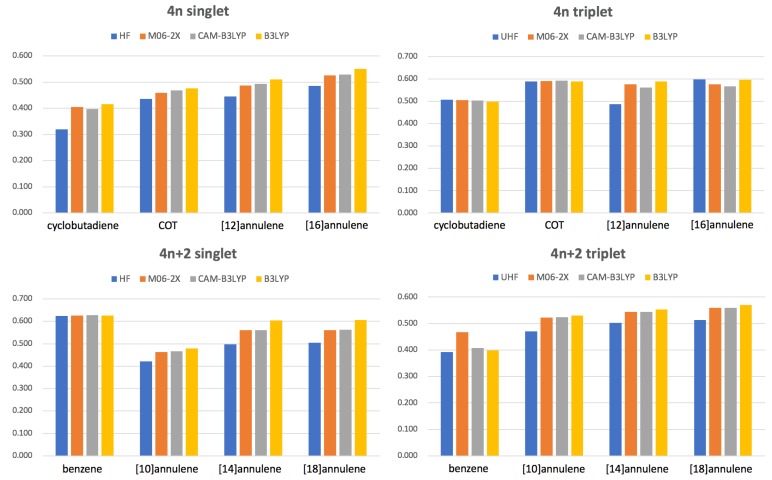
Values of $\bar{I_{\mathrm{ring}}}$ ${}^{1/N}$ for the lowest-lying states of the studied annulenes obtained with methods using a different percentage of Hartree Fock (HF) exchange: HF (100%), M06-2X (54%), CAM-B3LYP (19–65%) and B3LYP (19%).

$\bar{I_{\mathrm{ring}}}$${}^{1/N}$ values for different methods are collected in Figure 5. Our results confirm that in the large majority of cases B3LYP and HF are giving the most and the less aromatic species, respectively, among the DFA studied. In some cases (see, singlet benzene, triplet cyclobutadiene or triplet COT) there are no large differences among the DFAs because these species do not suffer from the delocalization error. However, in many other cases (see 4n+2 singlet annulenes larger than benzene) B3LYP clearly overestimates electron delocalization, sometimes even favoring a different geometrical arrangement of the atoms in the ring that permits larger electron delocalization (see Section 3.2). As expected, HF tends to overestimate electron localization whereas CAM-B3LYP and M06-2X provide very similar results lying between HF and B3LYP values. There is only two exceptions to this rule: the triplet states of benzene and [16]annulene. Unlike other DFAs and HF, the M06-2X geometry optimization of triplet benzene leads to a non-planar structure that does not correspond to the AQ structure [17] and, therefore, it is not as antiaromatic as one would expect. A careful examination of the potential energy surface of the triplet state of benzene confirms that M06-2X does not identify the AQ conformation as a minimum of energy at this level of theory. In the case of [16]annulene, the exception is that HF $\bar{I_{\mathrm{ring}}}$ ${}^{1/N}$ value is actually larger than that of CAM-B3LYP or M06-2X because, according to HF, the structure is more planar than predicted by these DFAs.

Interestingly, 4n+2 singlet molecules (with the exception of benzene) and 4n singlets tend to suffer the most from electron delocalization errors, whereas 4n+2 triplet molecules barely exhibit differences between B3LYP and other DFAs with higher percentage of HF exchange. The fact that M06-2X and CAM-B3LYP present very similar values for most molecules corroborates that it is actually the long-range part of Hartree-Fock exchange the one which is relevant to decrease the delocalization error [40]. In this sense, we recommend the use of a range-separation functional, such as CAM-B3LYP, to study aromatic and antiaromatic compounds.

## 4. Materials and Methods

The singlet ground state and the first triplet excited state of all the studied structures have been fully characterized. The optimizations have been performed with the Gaussian16 software package [99] using B3LYP [100,101], CAM-B3LYP [102], M06-2X [103] DFAs, and HF in combination with the 6-311G(d,p) basis set [104]. The harmonic vibrational frequencies were calculated at the corresponding level of theory in order to verify the nature of the stationary points of their potential energy surface (minima or transition states).

The calculation of the electronic aromaticity indices (AV1245, ${\mathrm{AV}}_{\min}$, BOA and FLU) uses a QTAIM atomic partition [105] performed by the AIMAll software [106]. The AOM resulting from the QTAIM partition and the molecular geometries are the input for the in-house ESI-3D program [27,31,107], which provides AV1245 [55], ${\mathrm{AV}}_{\min}$ [56], BLA, BOA, DIs [29,30], FLU [27], HOMA [33] (included in the Supplementary Material), $I_{\mathrm{ring}}$ [42], $\bar{I_{\mathrm{ring}}}$ and MCI [43] values. The numerical accuracy of the QTAIM calculations has been assessed using two criteria: (*i*) The integration of the Laplacian of the electron density ($\nabla^{2}\rho \left(r\right)$) within an atomic basin must be close to zero; (ii) the number of electrons in a molecule must be equal to the sum of all the electron populations of the molecule, and also equal to the sum of all the localization indices and half of the delocalization indices in the molecule. For all atomic calculations, integrated absolute values of $\nabla^{2}\rho \left(r\right)$ were always lower than 0.001 a.u. For all molecules, errors in the calculated number of electrons were always lower than 0.01 a.u. From our experience, these errors provide sufficient accuracy for all the indices here calculated except for the *normalized* multicenter indices of large rings, which require a numerical precision of the AOM well beyond the accuracy that one can obtain with AIMall or any other similar software available in the literature. For the latter and other reasons commented in Ref. [55], multicenter indices (MCI and $I_{\mathrm{ring}}$) cannot be used in large rings.

We have employed Mathematica [108] to perform all Hückel calculations, fittings and extrapolations presented in this manuscript.

## 5. Conclusions

In this paper, we have studied how the Hückel and Baird rules fade away in cyclic polyenes. According to pure HMO calculations and the seminal Baird study [17], antiaromatic annulenes lose their antiaromatic character at the same speed as the ring size increases, regardless of their multiplicity. However, a two-resonance parameter HMO method, permitting bond-order alternation, shows that the antiaromaticy of 4n singlets decreases more rapidly with the ring size. The conclusion is far less clear from calculations that consider the geometry relaxation because the potential energy surface of annulenes with more than eight carbon atoms often shows several configurations close in energy that display disparate aromatic characters. Nevertheless, density functional approximations reveal that the rules fade away much more quickly than it would be expected from the HMO method; they just do not follow a smooth trend.

The study of the level of theory employed in the calculation of annulenes reveals a clear tendency of density functional approximations with a low-percentage of HF exchange at long ranges to exhibit delocalization errors that lead to the overestimation of the aromatic character of the molecule and sometimes even to wrong geometries. Molecules with large ring structures are more prone to this kind of errors. These results are in line with previous findings [40,81,94] and suggest caution in choosing an appropriate density functional approximation to study aromatic and antiaromatic molecules. In particular, we recommend the use of a range-separation density functional approximation such as CAM-B3LYP.

## Figures and Tables

**Table 1:** Aromaticity indices for 4n+2 annulenes calculated with HF, B3LYP, CAM-B3LYP, and M06-2X and the 6-311G(d,p) basis set. ${}^{a}$$I_{\mathrm{ring}}$ values were too small for an accurate calculation of $I_{\mathrm{ring}}$ ^1/N^.

Structure	Multiplicity	Functional	FLU	$\bar{I_{\mathrm{ring}}}$ ^1/N^	BOA	BLA	$I_{\mathrm{ring}}$ ^1/N^	|${\mathrm{AV}}_{\min}$ |
C${}_{6}$H${}_{6}$	S	HF	0.000	0.624	0.000	0.000	0.597	10.25
		B3LYP	0.000	0.625	0.000	0.000	0.603	10.72
		CAM-B3LYP	0.000	0.628	0.000	0.000	0.603	10.71
		M06-2X	0.000	0.626	0.000	0.000	0.603	10.73
C${}_{6}$H${}_{6}$	T	HF	0.024	0.393	0.246	0.089	0.341	0.39
		B3LYP	0.025	0.399	0.275	0.090	0.353	1.51
		CAM-B3LYP	0.025	0.408	0.276	0.091	0.363	1.20
		M06-2X	0.041	0.467	0.281	0.056	0.380	0.28
C${}_{10}$H${}_{10}$ (twist)	S	HF	0.068	0.421	0.728	0.157	0.339	0.00
		B3LYP	0.052	0.480	0.639	0.128	0.322	0.03
		CAM-B3LYP	0.059	0.466	0.677	0.137	0.341	0.03
		M06-2X	0.058	0.463	0.670	0.136	0.325	0.05
C${}_{10}$H${}_{10}$ (heart)	S	HF	0.065	0.436	0.712	0.153	0.380	0.01
		B3LYP	0.000	0.610	0.007	0.009	0.579	5.19
		CAM-B3LYP	0.000	0.614	0.009	0.010	0.579	5.13
		M06-2X	0.000	0.611	0.010	0.010	0.579	5.11
C${}_{10}$H${}_{10}$ (naphthalene)	T	HF	0.030	0.470	0.364	0.085	0.353	0.14
		B3LYP	0.023	0.531	0.328	0.064	0.460	0.91
		CAM-B3LYP	0.027	0.524	0.364	0.072	0.446	0.68
		M06-2X	0.028	0.522	0.367	0.073	0.444	0.63
C${}_{10}$H${}_{10}$ (twist)	T	HF	0.020	0.478	0.266	0.068	0.295	0.05
		B3LYP	0.020	0.517	0.305	0.066	0.352	0.08
		CAM-B3LYP	0.022	0.513	0.324	0.071	0.347	0.07
		M06-2X	0.022	0.511	0.328	0.072	0.337	0.10
C${}_{14}$H${}_{14}$	S	HF	0.050	0.497	0.626	0.136	- ${}^{a}$	0.49
		B3LYP	0.001	0.605	0.010	0.008	- ${}^{a}$	4.24
		CAM-B3LYP	0.026	0.561	0.449	0.091	- ${}^{a}$	1.80
		M06-2X	0.025	0.561	0.438	0.088	- ${}^{a}$	1.89
C${}_{14}$H${}_{14}$ (TS)	S	CAM-B3LYP	0.000	0.609	0.007	0.007	-	4.29
		M06-2X	0.001	0.606	0.007	0.007	- ${}^{a}$	4.27
C${}_{14}$H${}_{14}$	T	HF	0.021	0.502	0.282	0.069	- ${}^{a}$	0.01
		B3LYP	0.017	0.554	0.302	0.060	- ${}^{a}$	0.13
		CAM-B3LYP	0.023	0.544	0.348	0.071	- ${}^{a}$	0.08
		M06-2X	0.022	0.544	0.349	0.071	- ${}^{a}$	0.04
C${}_{18}$H${}_{18}$	S	HF	0.049	0.504	0.616	0.133	0.472	0.57
		B3LYP	0.001	0.606	0.026	0.011	0.573	4.27
		CAM-B3LYP	0.026	0.563	0.446	0.090	0.530	1.80
		M06-2X	0.025	0.561	0.444	0.089	0.529	1.81
C${}_{18}$H${}_{18}$ (TS)	S	CAM-B3LYP	0.001	0.609	0.022	0.010	0.572	4.29
		M06-2X	0.001	0.607	0.022	0.010	0.572	4.28
C${}_{18}$H${}_{18}$	T	HF	0.018	0.514	0.257	0.060	0.411	0.12
		B3LYP	0.013	0.570	0.265	0.053	0.533	0.58
		CAM-B3LYP	0.019	0.559	0.324	0.066	0.513	0.30
		M06-2X	0.019	0.559	0.324	0.065	0.517	0.46

**Table 2:** Aromaticity indices for the studied 4n annulenes calculated with HF, B3LYP, CAM-B3LYP, and M06-2X and the 6-311G(d,p) basis set. ${}^{a}$$I_{\mathrm{ring}}$ values were too small for an accurate calculation of $I_{\mathrm{ring}}$ ^1/N^. ${}^{b}$${\mathrm{AV}}_{\min}$ cannot be calculated for rings with less than six members.

Structure	Multiplicity	Method	FLU	$\bar{I_{\mathrm{ring}}}$ ^1/N^	BOA	BLA	$I_{\mathrm{ring}}$ ^1/N^	|${\mathrm{AV}}_{\min}$ |
C${}_{4}$H${}_{4}$	S	HF	0.101	0.391	0.888	0.249	0.262	- ${}^{b}$
		B3LYP	0.104	0.416	0.900	0.247	0.266	- ${}^{b}$
		CAM-B3LYP	0.103	0.398	0.898	0.245	0.264	- ${}^{b}$
		M06-2X	0.103	0.405	0.898	0.242	0.268	- ${}^{b}$
C${}_{4}$H${}_{4}$	T	HF	0.010	0.507	0.000	0.000	0.433	- ${}^{b}$
		B3LYP	0.012	0.499	0.000	0.000	0.440	- ${}^{b}$
		CAM-B3LYP	0.011	0.504	0.000	0.000	0.439	- ${}^{b}$
		M06-2X	0.011	0.505	0.000	0.000	0.438	- ${}^{b}$
C${}_{8}$H${}_{8}$	S	HF	0.067	0.436	0.726	0.156	0.406	0.30
		B3LYP	0.056	0.477	0.664	0.134	0.441	0.72
		CAM-B3LYP	0.061	0.468	0.693	0.140	0.428	0.52
		M06-2X	0.062	0.460	0.694	0.141	0.427	0.51
C${}_{8}$H${}_{8}$	T	HF	0.001	0.590	0.000	0.000	0.534	4.07
		B3LYP	0.001	0.589	0.000	0.000	0.540	4.31
		CAM-B3LYP	0.001	0.593	0.000	0.000	0.539	4.29
		M06-2X	0.001	0.591	0.000	0.000	0.539	4.29
C${}_{12}$H${}_{12}$	S	HF	0.063	0.445	0.698	0.153	- ${}^{a}$	0.04
		B3LYP	0.042	0.511	0.565	0.115	- ${}^{a}$	0.01
		CAM-B3LYP	0.050	0.494	0.624	0.128	- ${}^{a}$	0.02
		M06-2X	0.050	0.488	0.624	0.128	- ${}^{a}$	0.06
C${}_{12}$H${}_{12}$	T	HF	0.021	0.487	0.280	0.067	- ${}^{a}$	0.07
		B3LYP	0.002	0.590	0.033	0.012	- ${}^{a}$	0.07
		CAM-B3LYP	0.015	0.562	0.288	0.056	- ${}^{a}$	0.21
		M06-2X	0.008	0.577	0.208	0.039	- ${}^{a}$	0.13
C${}_{16}$H${}_{16}$ (S${}_{4}$)	S	HF	0.054	0.486	0.651	0.139	0.440	0.33
		B3LYP	0.029	0.551	0.476	0.095	0.513	0.96
		CAM-B3LYP	0.041	0.529	0.564	0.113	0.484	0.64
		M06-2X	0.040	0.526	0.562	0.113	0.484	0.63
C${}_{16}$H${}_{16}$ (C${}_{1}$)	S	HF	0.053	0.488	0.643	0.139	0.452	0.25
		B3LYP	0.029	0.548	0.474	0.096	0.512	0.78
		CAM-B3LYP	0.040	0.530	0.555	0.113	0.487	0.55
		M06-2X	0.039	0.526	0.553	0.112	0.487	0.58
C${}_{16}$H${}_{16}$ (C${}_{s}$)	T	HF	0.001	0.598	0.013	0.005	- ${}^{a}$	1.08
		B3LYP	0.002	0.596	0.057	0.013	- ${}^{a}$	1.16
		CAM-B3LYP	0.015	0.568	0.294	0.059	- ${}^{a}$	0.35
		M06-2X	0.011	0.576	0.252	0.050	- ${}^{a}$	0.74

## Supplementary Materials

The Cartesian coordinates of the molecules as well as full tables and supplementary graphics are available online at https://www.mdpi.com/1420-3049/25/3/711/s1.

## Abbreviations

The following abbreviations are used in this manuscript:

AOM	Atomic overlaps matrix
AV1245	Aromaticity index for large rings [55]
${\mathrm{AV}}_{\min}$	Minimal value of 12-45 delocalizations [56]
BLA	Bond-length alternation
BOA	Bond-order alternation
DFA	Density Functional Approximation
DI	Delocalization index [28]
FLU	Fluctuation aromaticity index [27]
HF	Hartree-Fock
HMO	Hückel Molecular Orbital
HOMA	Harmonic Oscillator Model of Aromaticity [33]
LDA	Local density approximation [109]
MCI	Multicenter index [43]
RE	Resonance energy
TREPE	Topological resonance energy per π electron
$I_{\mathrm{ring}}$	Giambiagi’s multicenter index [42]
$\bar{I_{\mathrm{ring}}}$	Approximation to $I_{\mathrm{ring}}$ (Equation (8))

## References

[1] Von Ragué Schleyer P., Jiao H. (1996). What is aromaticity. Pure Appl. Chem..

[2] Feixas F., Matito E., Poater J., Solà M. (2015). Quantifying aromaticity with electron delocalisation measures. Chem. Soc. Rev..

[3] Solà M. (2017). Why aromaticity is a suspicious concept? Why?. Front. Chem..

[4] Boldyrev A.I., Wang L.S. (2005). All-metal aromaticity and antiaromaticity. Chem. Rev..

[5] Feixas F., Matito E., Poater J., Solà M. (2013). Metalloaromaticity. WIREs Comput. Mol. Sci..

[6] Wade K. (1971). The structural significance of the number of skeletal bonding electron-pairs in carboranes, the higher boranes and borane anions, and various transition-metal carbonyl cluster compounds. J. Chem. Soc. D: Chem. Common..

[7] Mingos D.M. (1984). Polyhedral Skeletal Electron Pair Approach. Acc. Chem. Res..

[8] Hirsch A., Chen Z., Jiao H. (2000). Spherical aromaticity in Ih symmetrical fullerenes: the 2 (*N* + 1) 2 rule. Angew. Chem. Int. Ed..

[9] Poater J., Solà M. (2011). Open-shell spherical aromaticity: The 2*N*^2^ + 2*N* + 1 (with *S* = *N* + 1/2) rule. Chem. Commun..

[10] Poater J., Solà M. (2019). Open-shell jellium aromaticity in metal clusters. Chem. Commun..

[11] Feixas F., Matito E., Poater J., Solà M. (2016). Rules of aromaticity. Applications of Topological Methods in Molecular Chemistry.

[12] Hückel E. (1931). Quantentheoretische Beitraäge zum Benzolproblem, I: Die Elektronenkonfiguration des Benzols und verwandter Verbindungen. Z. Physik.

[13] Hückel E. (1931). Quantentheoretische Beiträge zum Benzolproblem, II: Quantentheorie der induzierten Polaritäten. Z. Physik.

[14] Hückel E. (1932). Beiträge zum Problem der aromatischen und ungesättigten Verbingungen. III. Z. Physik.

[15] Hückel E. (1937). Grundzüge der Theorie ungesättigter und aromatischer Verbindungen. Z. Elektrochem..

[16] Gellini C., Salvi P.R. (2010). Structures of annulenes and model annulene systems in the ground and lowest excited states. Symmetry.

[17] Baird N.C. (1972). Quantum organic photochemistry. II. Resonance and aromaticity in the lowest 3. pi.. pi.* state of cyclic hydrocarbons. J. Am. Chem. Soc..

[18] Aihara J.I. (1978). Aromaticity-based theory of pericyclic reactions. Bull. Chem. Soc. Jpn..

[19] Ottosson H. (2012). Organic photochemistry: exciting excited-state aromaticity. Nat. Chem..

[20] Streifel B.C., Zafra J.L., Espejo G.L., Gómez-García C.J., Casado J., Tovar J.D. (2015). An Unusually Small Singlet–Triplet Gap in a Quinoidal 1, 6-Methano [10]annulene Resulting from Baird’s 4n *π*-Electron Triplet Stabilization. Angew. Chem. Int. Ed..

[21] Jorner K., Feixas F., Ayub R., Lindh R., Solà M., Ottosson H. (2016). Analysis of a compound class with triplet states stabilized by potentially Baird aromatic [10]annulenyl dicationic rings. Chem. Eur. J..

[22] Ueda M., Jorner K., Sung Y.M., Mori T., Xiao Q., Kim D., Ottosson H., Aida T., Itoh Y. (2017). Energetics of Baird aromaticity supported by inversion of photoexcited chiral [4*n*] annulene derivatives. Nat. Chem..

[23] Peeks M.D., Claridge T.D., Anderson H.L. (2017). Aromatic and antiaromatic ring currents in a molecular nanoring. Nature.

[24] Choi C.H., Kertesz M. (1998). Bond length alternation and aromaticity in large annulenes. J. Chem. Phys..

[25] Soncini A., Fowler P.W., Jenneskens L.W. (2004). Ring currents in large [4n + 2]-annulenes. Phys. Chem. Chem. Phys..

[26] Wannere C.S., Schleyer P.v.R. (2003). How Aromatic Are Large (4n + 2) *π* Annulenes?. Org. Lett..

[27] Matito E., Duran M., Solà M. (2005). The aromatic fluctuation index (FLU): A new aromaticity index based on electron delocalization. J. Chem. Phys..

[28] Bader R.F.W., Stephens M.E. (1974). Fluctuation and correlation of electrons in molecular systems. Chem. Phys. Lett..

[29] Bader R.F.W., Stephens M.E. (1975). Spatial localization of the electronic pair and number distributions in molecules. J. Am. Chem. Soc..

[30] Fradera X., Austen M.A., Bader R.F.W. (1999). The Lewis Model and Beyond. J. Phys. Chem. A.

[31] Matito E., Solà M., Salvador P., Duran M. (2007). Electron sharing indexes at the correlated level. Application to aromaticity calculations. Faraday Discuss..

[32] Mayer I. (1983). Charge, Bond Order, and Valence in the ab initio SCF Theory. Chem. Phys. Lett..

[33] Kruszewski J., Krygowski T.M. (1972). Definition of aromaticity basing on the harmonic oscillator model. Tetrahedron Lett..

[34] Krygowski T.M., Cyranski M.K. (2001). Structural aspects of aromaticity. Chem. Rev..

[35] Matito E., Poater J., Duran M., Solà M. (2005). An analysis of the changes in aromaticity and planarity along the reaction path of the simplest Diels–Alder reaction. Exploring the validity of different indicators of aromaticity. J. Mol. Struct. (Theochem).

[36] Feixas F., Matito E., Poater J., Solà M. (2008). On the performance of some aromaticity indices: A critical assessment using a test set. J. Comput. Chem..

[37] Fonseca Guerra C., Handgraaf J.W., Baerends E.J., Bickelhaupt F.M. (2004). Voronoi deformation density (VDD) charges: Assessment of the Mulliken, Bader, Hirshfeld, Weinhold, and VDD methods for charge analysis. J. Comput. Chem..

[38] Matito E., Poater J., Solà M., Duran M., Salvador P. (2005). Comparison of the AIM Delocalization Index and the Mayer and Fuzzy Atom Bond Orders. J. Phys. Chem. A.

[39] Matito E., Solà M., Duran M., Salvador P. (2006). Aromaticity Measures from Fuzzy-Atom Bond Orders (FBO). The Aromatic Fluctuation (FLU) and the para-Delocalization (PDI) Indexes. J. Phys. Chem. A.

[40] Casademont-Reig I., Woller T., Contreras-García J., Alonso M., Torrent-Sucarrat M., Matito E. (2018). New electron delocalization tools to describe the aromaticity in porphyrinoids. Phys. Chem. Chem. Phys..

[41] Giambiagi M., De Giambiagi M.S., Mundim K.C. (1990). Definition of a multicenter bond index. Struct. Chem..

[42] Giambiagi M., De Giambiagi M.S., Dos Santos Silva C.D., De Figuereido A.P. (2000). Multicenter bond indices as a measure of aromaticity. Phys. Chem. Chem. Phys..

[43] Bultinck P., Ponec R., Van Damme S. (2005). Multicenter bond indices as a new measure of aromaticity in polycyclic aromatic hydrocarbons. J. Phys. Org. Chem..

[44] Cioslowski J., Matito E., Solà M. (2007). Properties of Aromaticity Indices Based on the One-electron Density Matrix. J. Phys. Chem. A.

[45] Feixas F., Jiménez-Halla J., Matito E., Poater J., Solà M. (2010). A Test to Evaluate the Performance of Aromaticity Descriptors in All-Metal and Semimetal Clusters. An Appraisal of Electronic and Magnetic Indicators of Aromaticity. J. Chem. Theory Comput..

[46] Feixas F., Vandenbussche J., Bultinck P., Matito E., Solà M. (2011). Electron delocalization and aromaticity in low-lying excited states of archetypal organic compounds. Phys. Chem. Chem. Phys..

[47] Mercero J.M., Matito E., Ruipérez F., Infante I., Lopez X., Ugalde J.M. (2015). The Electronic Structure of the ${\mathrm{Al}}_{3}^{-}$ Anion: Is it Aromatic?. Chem. Eur. J..

[48] Fortenberry R.C., Novak C.M., Layfield J.P., Matito E., Lee T.J. (2018). Overcoming the Failure of Correlation for Out-of-Plane Motions in a Simple Aromatic: Rovibrational Quantum Chemical Analysis of c-C_3_H_2_. J. Chem. Theory Comput..

[49] Grande-Aztatzi R., Mercero J.M., Matito E., Frenking G., Ugalde J.M. (2017). The aromaticity of dicupra [10]annulenes. Phys. Chem. Chem. Phys..

[50] López R.V., Faza O.N., Matito E., López C.S. (2017). Cycloreversion of the CO_2_ trimer: A paradigmatic pseudopericyclic [2+ 2+ 2] cycloaddition reaction. Org. Biomol. Chem..

[51] Popov I.A., Pan F.X., You X.R., Li L.J., Matito E., Liu C., Zhai H.J., Sun Z.M., Boldyrev A.I. (2016). Peculiar All-Metal *σ*-Aromaticity of the [Au_2_Sb_16_]^4-^ Anion in the Solid State. Angew. Chem. Int. Ed..

[52] Min X., Popov I.A., Pan F.X., Li L.J., Matito E., Sun Z.M., Wang L.S., Boldyrev A.I. (2016). All-Metal Antiaromaticity in Sb_4_-Type Lanthanocene Anions. Angew. Chem. Int. Ed..

[53] Jiménez-Halla J.O.C., Matito E., Solà M., Braunschweig H., Hörl C., Krummenacher I., Wahler J. (2015). A theoretical study of the aromaticity in neutral and anionic borole compounds. Dalton Trans..

[54] Castro A.C., Osorio E., Cabellos J.L., Cerpa E., Matito E., Solà M., Swart M., Merino G. (2014). Exploring the Potential Energy Surface of E_2_P_4_ Clusters (E= Group 13 Element): The Quest for Inverse Carbon-Free Sandwiches. Chem. Eur. J..

[55] Matito E. (2016). Electronic Aromaticity Index for Large Rings. Phys. Chem. Chem. Phys..

[56] García-Fernández C., Sierda E., Abadia M., Bugenhagen B.E.C., Prosenc M.H., Wiesendanger R., Bazarnik M., Ortega J.E., Brede J., Matito E. (2017). Exploring the Relation Between Intramolecular Conjugation and Band Dispersion in One-Dimensional Polymers. J. Phys. Chem. C.

[57] Gutman I., Milun M., Trinajstić N. (1977). Graph theory and molecular orbitals. 19. Nonparametric resonance energies of arbitrary conjugated systems. J. Am. Chem. Soc..

[58] Feixas F., Matito E., Solà M., Poater J. (2008). Analysis of Hückel’s [4n+2] Rule through Electronic Delocalization Measures. J. Phys. Chem. A.

[59] Feixas F., Matito E., Solà M., Poater J. (2010). Patterns of *π*-electron delocalization in aromatic and antiaromatic organic compounds in the light of Hückel’s 4n+2 rule. Phys. Chem. Chem. Phys..

[60] Li X., Kuznetsov A.E., Zhang H.F., Boldyrev A.I., Wang L.S. (2001). Observation of all-metal aromatic molecules. Science.

[61] Jiménez-Halla J.O.C., Matito E., Blancafort L., Robles J., Solà M. (2009). Tuning aromaticity in trigonal alkaline earth metal clusters and their alkali metal salts. J. Comput. Chem..

[62] Garcia-Borràs M., Osuna S., Swart M., Luis J.M., Solà M. (2013). Maximum aromaticity as a guiding principle for the most suitable hosting cages in endohedral metallofullerenes. Angew. Chem. Int. Ed..

[63] Lu X., Chen Z. (2005). Curved pi-conjugation, aromaticity, and the related chemistry of small fullerenes. Chem. Rev..

[64] Osuka A., Saito S. (2011). Expanded porphyrins and aromaticity. Chem. Commun..

[65] Feixas F., Solà M., Swart M. (2009). Chemical bonding and aromaticity in metalloporphyrins 1, 2. Can. J. Chem..

[66] Sung Y.M., Oh J., Cha W.Y., Kim W., Lim J.M., Yoon M.C., Kim D. (2017). Control and switching of aromaticity in various all-aza-expanded porphyrins: spectroscopic and theoretical analyses. Chem. Rev..

[67] Tanaka T., Osuka A. (2015). Conjugated porphyrin arrays: synthesis, properties and applications for functional materials. Chem. Soc. Rev..

[68] Stępień M., Latos-Grażyński L., Sprutta N., Chwalisz P., Szterenberg L. (2007). Expanded porphyrin with a split personality: a Hückel–Möbius aromaticity switch. Angew. Chem. Int. Ed..

[69] Marcos E., Anglada J.M., Torrent-Sucarrat M. (2012). Theoretical study of the switching between Hückel and Möbius topologies for expanded porphyrins. J. Phys. Chem. C.

[70] Liu Z., Tian Z., Li W., Meng S., Wang L., Ma J. (2012). Chiral Interconversions of Pd and/or Au Bis-Metalated [32]Octaphyrins(1,0,1,0,1,0,1,0) Involving Hückel and Möbius Macrocyclic Topologies: A Theoretical Prediction. J. Org. Chem..

[71] Marcos E., Anglada J.M., Torrent-Sucarrat M. (2014). Effect of the Meso-Substituent in the Hückel-to-Möbius Topological Switches. J. Org. Chem..

[72] Alonso M., Geerlings P., De Proft F. (2014). Exploring the structure–aromaticity relationship in Hückel and Möbius N-fused pentaphyrins using DFT. Phys. Chem. Chem. Phys..

[73] Alonso M., Pinter B., Geerlings P., De Proft F. (2015). Metalated Hexaphyrins: From Understanding to Rational Design. Chem. Eur. J..

[74] Matito E., Feixas F., Solà M. (2007). Electron delocalization and aromaticity measures within the Hückel molecular orbital method. J. Mol. Struct. (Theochem).

[75] Karadakov P., Castaño O., Ftatev F., Enchev V. (1981). Some contributions and generalizations to the electronic theory of even polyenes and annulenes. Chem. Phys. Lett..

[76] Fratev F., Enchev V., Polansky O., Bonchev D. (1982). A theoretical—information study on the electron delocalization (aromaticity) of annulenes with and without bond alternation. J. Mol. Struct. (Theochem).

[77] Spitler E.L., Johnson C.A., Haley M.M. (2006). Renaissance of annulene chemistry. Chem. Rev..

[78] Koseki S., Toyota A. (1997). Energy component analysis of the Pseudo-Jahn-Teller effect in the ground and electronically excited states of the cyclic conjugated hydrocarbons: Cyclobutadiene, benzene, and cyclooctatetraene. J. Phys. Chem. A.

[79] Papadakis R., Ottosson H. (2015). The excited state antiaromatic benzene ring: A molecular Mr Hyde?. Chem. Soc. Rev..

[80] Moll J.F., Pemberton R.P., Gutierrez M.G., Castro C., Karney W.L. (2007). Configuration change in [14] annulene requires Möbius antiaromatic bond shifting. J. Am. Chem. Soc..

[81] Wannere C.S., Sattelmeyer K.W., Schaefer III H.F., Schleyer P.v.R. (2004). Aromaticity: The Alternating C-C Bond Length Structures of [14]-, [18]-, and [22] Annulene. Angew. Chem. Int. Ed..

[82] Feixas F., Solà M., Barroso J.M., Ugalde J.M., Matito E. (2014). New Approximation to the Third-Order Density. Application to the Calculation of Correlated Multicenter Indices. J. Chem. Theory Comput..

[83] Feixas F., Rodríguez-Mayorga M., Matito E., Solà M. (2015). Three-center bonding analyzed from correlated and uncorrelated third-order reduced density matrices. Comput. Theor. Chem..

[84] Mandado M., Graña A.M., Pérez-Juste I. (2008). Aromaticity in spin-polarized systems: Can rings be simultaneously alpha aromatic and beta antiaromatic?. J. Chem. Phys..

[85] Karadakov P.B. (2008). Aromaticity and antiaromaticity in the low-lying electronic states of cyclooctatetraene. J. Phys. Chem. A.

[86] Braten M.N., Castro C., Herges R., Köhler F., Karney W.L. (2008). The [12]annulene global minimum. J. Org. Chem..

[87] Castro C., Karney W.L. (2012). Mechanisms and Möbius strips: Understanding dynamic processes in annulenes. J. Phys. Org. Chem..

[88] Lee H.L., Li W.K. (2003). Computational study on the electrocyclic reactions of [16]annulene. Org. Biomol. Chem..

[89] Arbitman J.K., Michel C.S., Castro C., Karney W.L. (2019). Calculations Predict That Heavy-Atom Tunneling Dominates Möbius Bond Shifting in [12]-and [16] Annulene. Org. Lett..

[90] Merkle R., Savin A., Preuss H. (1992). Singly ionized first-row dimers and hydrides calculated with the fully-numerical density-functional program numol. J. Chem. Phys..

[91] Savin A., Seminario J.M. (1996). On degeneracy, near-degeneracy and density functional theory. Recent Developments of Modern Density Functional Theory.

[92] Cohen A.J., Mori-Sánchez P., Yang W. (2008). Insights into Current Limitations of Density Functional Theory. Science.

[93] Sancho-García J., Pérez-Jiménez A. (2007). Improved accuracy with medium cost computational methods for the evaluation of bond length alternation of increasingly long oligoacetylenes. Phys. Chem. Chem. Phys..

[94] Torrent-Sucarrat M., Navarro S., Cossío F.P., Anglada J.M., Luis J.M. (2017). Relevance of the DFT method to study expanded porphyrins with different topologies. J. Comput. Chem..

[95] Szczepanik D.W., Solà M., Andrzejak M., Pawełek B., Dominikowska J., Kukułka M., Dyduch K., Krygowski T.M., Szatylowicz H. (2017). The role of the long-range exchange corrections in the description of electron delocalization in aromatic species. J. Comput. Chem..

[96] Peccati F., Laplaza R., Contreras-García J. (2019). Overcoming Distrust in Solid State Simulations: Adding Error Bars to Computational Data. J. Phys. Chem. C.

[97] Sim E., Song S., Burke K. (2018). Quantifying density errors in DFT. J. Phys. Chem. Lett..

[98] Parr R.G., Yang W. (1989). Density-Functional Theory of Atoms and Molecules.

[99] Frisch M.J., Trucks G.W., Schlegel H.B., Scuseria G.E., Robb M.A., Cheeseman J.R., Scalmani G., Barone V., Petersson G.A., Nakatsuji H. (2016). Gaussian~16 Revision C.01.

[100] Becke A.D. (1993). Density-functional thermochemistry. III. The role of exact exchange. J. Chem. Phys..

[101] Stephens P.J., Devlin F.J., Chabalowski C.F., Frisch M.J. (1994). Ab initio calculation of vibrational absorption and circular dichroism spectra using density functional force fields. J. Phys. Chem..

[102] Yanai T., Tew D.P., Handy N.C. (2004). A new hybrid exchange–correlation functional using the Coulomb-attenuating method (CAM-B3LYP). Chem. Phys. Lett..

[103] Zhao Y., Truhlar D.G. (2008). The M06 suite of density functionals for main group thermochemistry, thermochemical kinetics, noncovalent interactions, excited states, and transition elements: Two new functionals and systematic testing of four M06-class functionals and 12 other functionals. Theor. Chem. Acc..

[104] Krishnan R., Binkley J.S., Seeger R., Pople J.A. (1980). Self-consistent molecular orbital methods. XX. A basis set for correlated wave functions. J. Chem. Phys..

[105] Bader R.F.W. (1990). Atoms in Molecules: A Quantum Theory.

[106] Keith T.A. (2014). AIMAll (Version 14.11.23).

[107] Matito E. (2015). ESI-3D.

[108] Wolfram S. (2014). Mathematica 10.

[109] Vosko S.H., Wilk L., Nusair M. (1980). Accurate spin-dependent electron liquid correlation energies for local spin density calculations: A critical analysis. Can. J. Phys..

