# Supplementary material for: How do the Hückel and Baird Rules Fade away in Annulenes?
Source: Molecules. 2020 Feb 7;25(3):711. doi: 10.3390/molecules25030711 (PMC7037833; doi:10.3390/molecules25030711)
Supplement: Supplementary file 1 [file molecules-25-00711-s001.zip › SI-Geometries.pdf]

Supporting information:

Complete optimized geometries (in Å) of the studied C_nH_n annulene series (n=4-18, even number) using B3LYP, CAM-B3LYP, M06-2X density functional approximations and HF in combination with the 6-311G(d,p) basis set.

Table 1. C_4H_4 singlet optimized with HF:

6	0.000000	0.658824	0.783126
6	0.000000	0.658824	-0.783126
6	0.000000	-0.658824	0.783126
6	0.000000	-0.658824	-0.783126
1	0.000000	1.419468	1.538886
1	0.000000	1.419468	-1.538886
1	0.000000	-1.419468	1.538886
1	0.000000	-1.419468	-1.538886

Table 2. C_4H_4 singlet optimized with B3LYP:

6	0.000000	0.665797	0.789329
6	0.000000	0.665797	-0.789329
6	0.000000	-0.665797	0.789329
6	0.000000	-0.665797	-0.789329
1	0.000000	1.432935	1.551186
1	0.000000	1.432935	-1.551186
1	0.000000	-1.432935	1.551186
1	0.000000	-1.432935	-1.551186

Table 3. C_4H_4 singlet optimized with CAM-B3LYP:

6	0.000000	0.662972	0.785234
6	0.000000	0.662972	-0.785234
6	0.000000	-0.662972	0.785234
6	0.000000	-0.662972	-0.785234
1	0.000000	1.428243	1.547651
1	0.000000	1.428243	-1.547651
1	0.000000	-1.428243	1.547651
1	0.000000	-1.428243	-1.547651

Table 4. C_4H_4 singlet optimized with M06-2X:

6	0.000000	0.663994	0.785233
6	0.000000	0.663994	-0.785233
6	0.000000	-0.663994	0.785233
6	0.000000	-0.663994	-0.785233
1	0.000000	1.430282	1.547110
1	0.000000	1.430282	-1.547110
1	0.000000	-1.430282	1.547110

1	0.000000	-1.430282	-1.547110

Table 5. C_4H_4 triplet optimized with UHF:

6	0.000000	0.713598	0.713615
6	0.000000	0.713598	-0.713615
6	0.000000	-0.713598	0.713615
6	0.000000	-0.713598	-0.713615
1	0.000000	1.471526	1.470818
1	0.000000	1.471526	-1.470818
1	0.000000	-1.471526	1.470818
1	0.000000	-1.471526	-1.470818

Table 6. C_4H_4 triplet optimized with UB3LYP:

6	0.000000	0.719753	0.719835
6	0.000000	0.719753	-0.719835
6	0.000000	-0.719753	0.719835
6	0.000000	-0.719753	-0.719835
1	0.000000	1.484200	1.482506
1	0.000000	1.484200	-1.482506
1	0.000000	-1.484200	1.482506
1	0.000000	-1.484200	-1.482506

Table 7. C_4H_4 triplet optimized with UCAM-B3LYP:

6	0.000000	0.716472	0.716523
6	0.000000	0.716472	-0.716523
6	0.000000	-0.716472	0.716523
6	0.000000	-0.716472	-0.716523
1	0.000000	1.480218	1.478803
1	0.000000	1.480218	-1.478803
1	0.000000	-1.480218	1.478803
1	0.000000	-1.480218	-1.478803

Table 8. C_4H_4 triplet optimized with UM06-2X:

6	0.000000	0.716687	0.716738
6	0.000000	0.716687	-0.716738
6	0.000000	-0.716687	0.716738
6	0.000000	-0.716687	-0.716738
1	0.000000	1.480318	1.478820
1	0.000000	1.480318	-1.478820
1	0.000000	-1.480318	1.478820
1	0.000000	-1.480318	-1.478820

Table 9. C_6H_6 singlet optimized with HF:

6	1.066021	-0.884627	0.000161

6	-0.233139	-1.365560	-0.000275
6	1.299241	0.480811	-0.000061
1	-0.414127	-2.425843	-0.000362
1	2.307988	0.854157	-0.000469
6	-1.299181	-0.480924	0.000073
6	0.233075	1.365602	0.000005
1	-2.307923	-0.854281	-0.000027
1	0.414175	2.425857	-0.000413
6	-1.066019	0.884720	0.000189
1	-1.893775	1.571568	-0.000133
1	1.893671	-1.571592	0.000857

Table 10. C_6H_6 singlet optimized with B3LYP:

6	0.000000	0.000000	1.393968
6	0.000000	1.207156	0.696959
6	0.000000	-1.207156	0.696959
1	0.000000	2.146302	1.239153
1	0.000000	-2.146302	1.239153
6	0.000000	1.207156	-0.696959
6	0.000000	-1.207156	-0.696959
1	0.000000	2.146302	-1.239153
1	0.000000	-2.146302	-1.239153
6	0.000000	0.000000	-1.393968
1	0.000000	0.000000	-2.478381
1	0.000000	0.000000	2.478381

Table 11. C_6H_6 singlet optimized with CAM-B3LYP:

6	0.000000	0.000000	1.388255
6	0.000000	1.202218	0.694113
6	0.000000	-1.202218	0.694113
1	0.000000	2.140653	1.235888
1	0.000000	-2.140653	1.235888
6	0.000000	1.202218	-0.694113
6	0.000000	-1.202218	-0.694113
1	0.000000	2.140653	-1.235888
1	0.000000	-2.140653	-1.235888
6	0.000000	0.000000	-1.388255
1	0.000000	0.000000	-2.471847
1	0.000000	0.000000	2.471847

Table 12. C_6H_6 singlet optimized with M06-2X:

6	0.000000	0.000000	1.390881
6	0.000000	1.204750	0.695536
6	0.000000	-1.204750	0.695536
1	0.000000	2.142940	1.237639

1	0.000000	-2.142940	1.237639
6	0.000000	1.204750	-0.695536
6	0.000000	-1.204750	-0.695536
1	0.000000	2.142940	-1.237639
1	0.000000	-2.142940	-1.237639
6	0.000000	0.000000	-1.390881
1	0.000000	0.000000	-2.474377
1	0.000000	0.000000	2.474377

Table 13. C_6H_6 triplet optimized with UHF:

6	0.000000	1.208117	0.760615
6	0.000000	1.208117	-0.760615
6	0.000000	0.000000	1.442394
1	0.000000	2.145858	-1.283154
1	0.000000	0.000000	2.518776
6	0.000000	0.000000	-1.442394
6	0.000000	-1.208117	0.760615
1	0.000000	0.000000	-2.518776
1	0.000000	-2.145858	1.283154
6	0.000000	-1.208117	-0.760615
1	0.000000	-2.145858	-1.283154
1	0.000000	2.145858	1.283154

Table 14. C_6H_6 triplet optimized with UB3LYP:

6	0.000000	1.207499	0.760333
6	0.000000	1.207499	-0.760333
6	0.000000	0.000000	1.440821
1	0.000000	2.152536	-1.286982
1	0.000000	0.000000	2.526293
6	0.000000	0.000000	-1.440821
6	0.000000	-1.207499	0.760333
1	0.000000	0.000000	-2.526293
1	0.000000	-2.152536	1.286982
6	0.000000	-1.207499	-0.760333
1	0.000000	-2.152536	-1.286982
1	0.000000	2.152536	1.286982

Table 15. C_6H_6 triplet optimized with UCAM-B3LYP:

6	0.000000	1.202354	0.758354
6	0.000000	1.202354	-0.758354
6	0.000000	0.000000	1.436639
1	0.000000	2.146740	-1.284677
1	0.000000	0.000000	2.521141
6	0.000000	0.000000	-1.436639
6	0.000000	-1.202354	0.758354

1	0.000000	0.000000	-2.521141
1	0.000000	-2.146740	1.284677
6	0.000000	-1.202354	-0.758354
1	0.000000	-2.146740	-1.284677
1	0.000000	2.146740	1.284677

Table 16. C_6H_6 triplet optimized with UM06-2X:

6	-0.088861	-0.692927	1.257780
6	0.281403	-1.388247	0.000000
6	-0.088861	0.658915	1.242931
1	1.271629	-1.852458	0.000000
1	-0.265966	1.223470	2.152189
6	-0.088861	-0.692927	-1.257780
6	0.040749	1.367153	0.000000
1	-0.294958	-1.254275	-2.161927
1	0.049981	2.448781	0.000000
6	-0.088861	0.658915	-1.242931
1	-0.265966	1.223470	-2.152189
1	-0.294958	-1.254275	2.161927

Table 17. C_8H_8 singlet optimized with HF:

6	-1.691292	-0.030129	0.380768
6	-1.196113	1.196113	0.380770
6	-0.030129	1.691292	-0.380768
6	1.196113	1.196113	-0.380770
6	1.691292	0.030129	0.380768
6	1.196113	-1.196113	0.380770
6	0.030129	-1.691292	-0.380768
6	-1.196113	-1.196113	-0.380770
1	-2.591945	-0.207778	0.948316
1	-1.720853	1.949360	0.948320
1	-0.207778	2.591945	-0.948316
1	1.949360	1.720853	-0.948320
1	2.591945	0.207778	0.948316
1	1.720853	-1.949360	0.948320
1	0.207778	-2.591945	-0.948316
1	-1.949360	-1.720853	-0.948320

Table 18. C_8H_8 singlet optimized with B3LYP:

6	-1.701296	-0.038873	0.375227
6	-1.203335	1.203335	0.375047
6	-0.038873	1.701296	-0.375227
6	1.203335	1.203335	-0.375047
6	1.701296	0.038873	0.375227
6	1.203335	-1.203335	0.375047

6	0.038873	-1.701296	-0.375227
6	-1.203335	-1.203335	-0.375047
1	-2.622600	-0.210187	0.929995
1	-1.751175	1.963731	0.929704
1	-0.210187	2.622600	-0.929995
1	1.963731	1.751175	-0.929704
1	2.622600	0.210187	0.929995
1	1.751175	-1.963731	0.929704
1	0.210187	-2.622600	-0.929995
1	-1.963731	-1.751175	-0.929704

Table 19. C_8H_8 singlet optimized with CAM-B3LYP:

6	1.193495	1.193495	0.385388
6	-0.042144	1.687349	0.385324
6	-1.193495	1.193495	-0.385388
6	-1.687349	-0.042144	-0.385324
6	-1.193495	-1.193495	0.385388
6	0.042144	-1.687349	0.385324
6	1.193495	-1.193495	-0.385388
6	1.687349	0.042144	-0.385324
1	1.950323	1.724335	0.958480
1	-0.224657	2.593616	0.958391
1	-1.724335	1.950323	-0.958480
1	-2.593616	-0.224657	-0.958391
1	-1.950323	-1.724335	0.958480
1	0.224657	-2.593616	0.958391
1	1.724335	-1.950323	-0.958480
1	2.593616	0.224657	-0.958391

Table 20. C_8H_8 singlet optimized with M06-2X:

6	-1.681508	-0.049972	0.397541
6	-1.189539	1.189539	0.397611
6	-0.049972	1.681508	-0.397541
6	1.189539	1.189539	-0.397611
6	1.681508	0.049972	0.397541
6	1.189539	-1.189539	0.397611
6	0.049972	-1.681508	-0.397541
6	-1.189539	-1.189539	-0.397611
1	-2.578317	-0.242619	0.982279
1	-1.710189	1.944597	0.982466
1	-0.242619	2.578317	-0.982279
1	1.944597	1.710189	-0.982466
1	2.578317	0.242619	0.982279
1	1.710189	-1.944597	0.982466
1	0.242619	-2.578317	-0.982279
1	-1.944597	-1.710189	-0.982466

Table 21. C_8H_8 triplet optimized with UHF:

6	0.697034	1.682964	0.000075
6	-0.697160	1.682911	0.000074
6	-1.682964	0.697034	-0.000075
6	-1.682911	-0.697160	-0.000074
6	-0.697034	-1.682964	0.000075
6	0.697160	-1.682911	0.000074
6	1.682964	-0.697034	-0.000075
6	1.682911	0.697160	-0.000074
1	1.109237	2.678272	0.000147
1	-1.109437	2.678189	0.000146
1	-2.678272	1.109237	-0.000147
1	-2.678189	-1.109437	-0.000146
1	-1.109237	-2.678272	0.000147
1	1.109437	-2.678189	0.000146
1	2.678272	-1.109237	-0.000147
1	2.678189	1.109437	-0.000146

Table 22. C_8H_8 triplet optimized with UB3LYP:

6	0.701414	1.693427	0.000092
6	-0.701540	1.693375	0.000091
6	-1.693427	0.701414	-0.000092
6	-1.693375	-0.701540	-0.000091
6	-0.701414	-1.693427	0.000092
6	0.701540	-1.693375	0.000091
6	1.693427	-0.701414	-0.000092
6	1.693375	0.701540	-0.000091
1	1.116885	2.697808	0.001165
1	-1.117088	2.697724	0.001164
1	-2.697808	1.116885	-0.001165
1	-2.697724	-1.117087	-0.001164
1	-1.116885	-2.697808	0.001165
1	1.117087	-2.697724	0.001164
1	2.697808	-1.116885	-0.001165
1	2.697724	1.117087	-0.001164

Table 23. C_8H_8 triplet optimized with UCAM-B3LYP:

6	0.698288	1.685934	0.000318
6	-0.698414	1.685881	0.000317
6	-1.685934	0.698288	-0.000318
6	-1.685881	-0.698414	-0.000317
6	-0.698288	-1.685934	0.000318
6	0.698414	-1.685881	0.000317
6	1.685934	-0.698288	-0.000318
6	1.685881	0.698414	-0.000317
1	1.113681	2.689197	0.000698
1	-1.113882	2.689113	0.000698
1	-2.689196	1.113681	-0.000698
1	-2.689113	-1.113882	-0.000698
1	-1.113681	-2.689196	0.000698

1	1.113882	-2.689113	0.000698
1	2.689196	-1.113681	-0.000698
1	2.689113	1.113882	-0.000698

Table 24. C_8H_8 triplet optimized with UM06-2X:

6	0.699878	1.689622	-0.000108
6	-0.700004	1.689570	-0.000108
6	-1.689622	0.699878	0.000108
6	-1.689570	-0.700004	0.000108
6	-0.699878	-1.689622	-0.000108
6	0.700004	-1.689570	-0.000108
6	1.689622	-0.699878	0.000108
6	1.689570	0.700004	0.000108
1	1.114901	2.693223	0.001232
1	-1.115103	2.693140	0.001231
1	-2.693223	1.114901	-0.001232
1	-2.693140	-1.115103	-0.001231
1	-1.114901	-2.693223	0.001232
1	1.115103	-2.693140	0.001231
1	2.693223	-1.114901	-0.001232
1	2.693140	1.115103	-0.001231

Table 25. $C_{10}H_{10}$ singlet twist isomer optimized with HF:

6	0.337834	-1.441633	-0.566733
6	-1.776604	-1.134666	0.648230
6	-2.292785	-0.044047	0.091891
6	-1.604216	0.959546	-0.762792
6	-0.456813	1.598564	-0.589095
1	0.197828	-1.463835	1.502648
1	-2.416840	-1.761687	1.247642
1	-3.351859	0.127522	0.211691
1	-2.147123	1.192630	-1.666728
1	-0.158120	2.272130	-1.378510
6	-0.337178	-1.441456	0.566660
6	1.777161	-1.134279	-0.648100
6	2.292732	-0.043327	-0.091824
6	1.603687	0.960046	0.762656
6	0.456079	1.598712	0.588984
1	-0.197017	-1.464647	-1.502786
1	2.418042	-1.761130	-1.246998
1	3.351901	0.128461	-0.211186
1	2.146406	1.193441	1.666650
1	0.157406	2.272361	1.378319

Table 26. $C_{10}H_{10}$ singlet twist isomer optimized with B3LYP:

6	0.339395	-1.421621	-0.577402
6	-1.765131	-1.107127	0.678590

6	-2.300949	-0.025433	0.077887
6	-1.607582	0.945371	-0.791065
6	-0.427374	1.556254	-0.605219
1	0.216618	-1.462585	1.512493
1	-2.399760	-1.708252	1.325925
1	-3.372421	0.134643	0.189727
1	-2.137476	1.176058	-1.714729
1	-0.059999	2.173792	-1.424196
6	-0.339214	-1.421480	0.577440
6	1.765252	-1.107067	-0.678535
6	2.300937	-0.025283	-0.077845
6	1.607434	0.945460	0.791029
6	0.427209	1.556309	0.605134
1	-0.216376	-1.463217	-1.512459
1	2.399965	-1.708054	-1.325910
1	3.372402	0.135004	-0.189649
1	2.137295	1.176346	1.714669
1	0.059900	2.173970	1.424044

Table 27. $C_{10}H_{10}$ singlet twist isomer optimized with CAM-B3LYP:

6	0.334085	-1.414445	-0.575338
6	-1.760811	-1.103810	0.675632
6	-2.289206	-0.026898	0.080534
6	-1.592213	0.936418	-0.794990
6	-0.425300	1.553879	-0.606037
1	0.224535	-1.442965	1.508026
1	-2.395858	-1.709894	1.315630
1	-3.357100	0.144051	0.197380
1	-2.115082	1.151534	-1.724722
1	-0.061844	2.179151	-1.419018
6	-0.333333	-1.414266	0.575265
6	1.761455	-1.103399	-0.675500
6	2.289172	-0.026125	-0.080397
6	1.591601	0.936951	0.794845
6	0.424471	1.554041	0.605876
1	-0.223661	-1.443814	-1.508135
1	2.397129	-1.709278	-1.315061
1	3.357064	0.145260	-0.196839
1	2.114306	1.152540	1.724583
1	0.060996	2.179338	1.418814

Table 28. $C_{10}H_{10}$ singlet twist isomer optimized with M06-2X:

6	0.326469	-1.401045	-0.581352
6	-1.754425	-1.082293	0.692727
6	-2.284041	-0.012962	0.075357
6	-1.575816	0.922321	-0.825872

6	-0.399935	1.524891	-0.623361
1	0.244839	-1.427481	1.50686
1	-2.386454	-1.670604	1.352026
1	-3.350257	0.167044	0.195031
1	-2.078991	1.113240	-1.771627
1	0.010308	2.114351	-1.440955
6	-0.326699	-1.401002	0.581355
6	1.754198	-1.082405	-0.692751
6	2.284065	-0.013224	-0.075317
6	1.575994	0.922136	0.825952
6	0.400236	1.524887	0.623314
1	-0.245056	-1.427543	-1.506862
1	2.386001	-1.670618	-1.352362
1	3.350378	0.166651	-0.195147
1	2.079029	1.112839	1.771822
1	-0.010065	2.114297	1.440913

Table 29. $C_{10}H_{10}$ singlet heart isomer optimized with HF:

6	0.016071	1.700736	-0.525203
1	0.082150	-0.641985	1.106809
6	-1.273484	1.384799	-0.543379
6	-2.214757	0.563995	0.273218
6	-2.276086	-0.760915	0.369324
6	-1.184514	-1.609493	-0.145648
1	-1.815002	1.853482	-1.354220
1	-3.047869	1.132490	0.659864
1	-3.151019	-1.209284	0.812666
1	-1.382613	-2.309017	-0.942597
6	0.032247	-1.323474	0.281580
6	1.157929	1.521932	0.422951
6	2.130767	0.618552	0.479994
6	2.280111	-0.670448	-0.234627
6	1.333864	-1.599795	-0.318729
1	1.286291	2.383866	1.062204
1	2.945356	0.846781	1.150906
1	3.250545	-0.859852	-0.664195
1	1.510523	-2.514163	-0.859157
1	0.308749	2.362349	-1.329155

Table 30. $C_{10}H_{10}$ singlet heart isomer optimized with B3LYP:

6	-0.138604	1.952192	0.000000
1	0.538690	-0.187818	0.000000
6	-0.071357	1.583597	1.373438
6	0.012800	0.533366	2.313746
6	0.012800	-0.868800	2.339892
6	0.032025	-1.744653	1.258600

1	-0.122413	2.511436	1.939344
1	0.026859	0.943797	3.320557
1	-0.061781	-1.302373	3.333225
1	-0.154341	-2.803506	1.412903
6	0.224833	-1.216418	0.000000
6	-0.071357	1.583597	-1.373438
6	0.012800	0.533366	-2.313746
6	0.012800	-0.868800	-2.339892
6	0.032025	-1.744653	-1.258600
1	-0.122413	2.511436	-1.939344
1	0.026859	0.943797	-3.320557
1	-0.061781	-1.302373	-3.333225
1	-0.154341	-2.803506	-1.412903
1	-0.267931	3.032346	0.000000

Table 31. $C_{10}H_{10}$ singlet heart isomer optimized with CAM-B3LYP:

6	-0.148038	1.937638	0.000000
1	0.535225	-0.171971	0.000000
6	-0.074532	1.572847	1.368132
6	0.018054	0.529669	2.305912
6	0.018054	-0.866739	2.331525
6	0.031243	-1.733198	1.251292
1	-0.128849	2.500193	1.932139
1	0.036254	0.940628	3.311214
1	-0.051279	-1.302010	3.323448
1	-0.152078	-2.792686	1.398133
6	0.219770	-1.198711	0.000000
6	-0.074532	1.572847	-1.368132
6	0.018054	0.529669	-2.305912
6	0.018054	-0.866739	-2.331525
6	0.031243	-1.733198	-1.251292
1	-0.128849	2.500193	-1.932139
1	0.036254	0.940628	-3.311214
1	-0.051279	-1.302010	-3.323448
1	-0.152078	-2.792686	-1.398133
1	-0.287540	3.015217	0.000000

Table 32. $C_{10}H_{10}$ singlet heart isomer optimized with M06-2X:

6	-0.192515	1.928333	0.000000
1	0.602438	-0.168018	0.000000
6	-0.093619	1.568677	1.370308
6	0.033767	0.529098	2.311736
6	0.033767	-0.869760	2.338087
6	0.033819	-1.727623	1.248059
1	-0.165315	2.496280	1.932808
1	0.065781	0.943078	3.315740

1	-0.034360	-1.307835	3.329007
1	-0.173017	-2.785044	1.379482
6	0.241833	-1.183191	0.000000
6	-0.093619	1.568677	-1.370308
6	0.033767	0.529098	-2.311736
6	0.033767	-0.869760	-2.338087
6	0.033819	-1.727623	-1.248059
1	-0.165315	2.496280	-1.932808
1	0.065781	0.943078	-3.315740
1	-0.034360	-1.307835	-3.329007
1	-0.173017	-2.785044	-1.379482
1	-0.377340	2.999513	0.000000

Table 33. $C_{10}H_{10}$ triplet naphthalene isomer optimized with UHF:

6	2.275974	-0.723700	-0.186449
6	2.276018	0.723660	0.186345
6	1.258540	1.605194	-0.010421
6	-0.000206	1.251749	-0.610209
6	-1.258425	1.605130	-0.009689
6	-2.276049	0.723497	0.187142
6	-2.276027	-0.723522	-0.187237
6	-1.258423	-1.605138	0.009742
6	-0.000222	-1.251645	0.610239
6	1.258533	-1.605225	0.010553
1	3.162218	-1.078371	-0.686991
1	3.162385	1.078338	0.686664
1	1.359227	2.588742	0.422357
1	-0.000379	0.408085	-1.270598
1	-1.358533	2.588268	0.424173
1	-3.161767	1.077547	0.689033
1	-3.161726	-1.077608	-0.689136
1	-1.358512	-2.588335	-0.423991
1	-0.000406	-0.407793	1.270401
1	1.359219	-2.588864	-0.422021

Table 34. $C_{10}H_{10}$ triplet naphthalene isomer optimized with UB3LYP:

6	2.329884	-0.718439	-0.137951
6	2.329897	0.718399	0.137955
6	1.268856	1.569237	-0.004729
6	-0.000140	1.169619	-0.535539
6	-1.268589	1.568975	-0.003683
6	-2.330060	0.718384	0.138496
6	-2.330070	-0.718361	-0.138516
6	-1.268625	-1.568969	0.003707
6	-0.000155	-1.169596	0.535525
6	1.268818	-1.569254	0.004735

1	3.267522	-1.136104	-0.495965
1	3.267551	1.136056	0.495936
1	1.370876	2.581606	0.383394
1	-0.000376	0.369691	-1.264105
1	-1.369842	2.580929	0.385749
1	-3.267568	1.135957	0.496903
1	-3.267588	-1.135927	-0.496906
1	-1.369906	-2.580949	-0.385650
1	-0.000378	-0.369613	1.264032
1	1.370809	-2.581622	-0.383399

Table 35. $C_{10}H_{10}$ triplet naphthalene isomer optimized with UCAM-B3LYP:

6	2.311449	-0.717932	-0.147100
6	2.311459	0.717917	0.147077
6	1.262221	1.565453	-0.006479
6	-0.000121	1.171199	-0.550721
6	-1.262117	1.565246	-0.006051
6	-2.311535	0.717895	0.147533
6	-2.311532	-0.717900	-0.147558
6	-1.262122	-1.565248	0.006067
6	-0.000124	-1.171169	0.550720
6	1.262215	-1.565464	0.006515
1	3.241715	-1.126326	-0.530982
1	3.241750	1.126311	0.530897
1	1.360824	2.576478	0.383267
1	-0.000003	0.360433	-1.265862
1	-1.360439	2.576037	0.384415
1	-3.241485	1.126208	0.532205
1	-3.241483	-1.126221	-0.532220
1	-1.360444	-2.576058	-0.384352
1	-0.000008	-0.360335	1.265788
1	1.360815	-2.576511	-0.383175

Table 36. $C_{10}H_{10}$ triplet naphthalene isomer optimized with UM06-2X:

6	2.312791	-0.717429	-0.151362
6	2.312778	0.717415	0.151462
6	1.259832	1.562535	-0.006378
6	-0.000052	1.163093	-0.562636
6	-1.259718	1.562377	-0.006178
6	-2.312865	0.717429	0.151633
6	-2.312899	-0.717375	-0.151556
6	-1.259762	-1.562360	0.006146
6	-0.000068	-1.163153	0.562593
6	1.259790	-1.562533	0.006276
1	3.241142	-1.127274	-0.538228
1	3.241078	1.127252	0.538458

1	1.348527	2.574367	0.384155
1	0.000032	0.355086	-1.283134
1	-1.348084	2.573996	0.385015
1	-3.240960	1.127328	0.539004
1	-3.241020	-1.127229	-0.538914
1	-1.348169	-2.573945	-0.385122
1	0.000038	-0.355274	1.283228
1	1.348455	-2.574289	-0.384458

Table 37. $C_{10}H_{10}$ triplet twist optimized with UHF:

6	0.409205	-1.563695	-0.621913
6	-1.622343	-0.959496	0.834352
6	-2.264536	-0.000056	0.000312
6	-1.622379	0.959284	-0.834260
6	-0.409398	1.563978	-0.621770
1	-0.078400	-2.268275	1.369657
1	-2.186852	-1.307669	1.685526
1	-3.343096	0.000330	0.000714
1	-2.186962	1.307037	-1.685552
1	-0.078892	2.268341	-1.369889
6	-0.409115	-1.563908	0.621657
6	1.622251	-0.959207	-0.834565
6	2.264567	-0.000285	0.000206
6	1.622364	0.959229	0.834413
6	0.409476	1.564061	0.621583
1	0.078179	-2.267561	-1.370258
1	2.186536	-1.306711	-1.686163
1	3.343084	-0.000743	0.000727
1	2.186853	1.307126	1.685703
1	0.078991	2.268696	1.369453

Table 38. $C_{10}H_{10}$ triplet twist optimized with UB3LYP:

6	0.398174	-1.479928	-0.623724
6	-1.633946	-0.935233	0.836243
6	-2.296540	0.000017	-0.000043
6	-1.633919	0.935280	-0.836231
6	-0.398145	1.479980	-0.623668
1	-0.003275	-2.126932	1.406729
1	-2.180435	-1.290684	1.708104
1	-3.383575	-0.000034	-0.000124
1	-2.180357	1.290786	-1.708100
1	-0.003218	2.126914	-1.406696
6	-0.398203	-1.479971	0.623729
6	1.633909	-0.935200	-0.836319
6	2.296583	-0.000001	-0.000015
6	1.633931	0.935163	0.836308

6	0.398198	1.479904	0.623737
1	0.003164	-2.126809	-1.406757
1	2.180285	-1.290581	-1.708277
1	3.383614	-0.000021	-0.000065
1	2.180315	1.290541	1.708264
1	0.003234	2.126751	1.406822

Table 39. $C_{10}H_{10}$ triplet twist optimized with UCAM-B3LYP:

6	0.393930	-1.470200	-0.625623
6	-1.619772	-0.924146	0.842776
6	-2.283161	0.001244	0.000161
6	-1.617982	0.924336	-0.844818
6	-0.392442	1.469348	-0.626440
1	0.003485	-2.120406	1.402654
1	-2.160842	-1.268780	1.720743
1	-3.368952	0.003452	0.001665
1	-2.157386	1.266692	-1.724698
1	0.008384	2.116247	-1.404521
6	-0.393955	-1.470399	0.625400
6	1.619645	-0.923957	-0.842958
6	2.283227	0.001014	0.000264
6	1.618074	0.924263	0.844892
6	0.392508	1.469342	0.626348
1	-0.003716	-2.119821	-1.403108
1	2.160500	-1.268033	-1.721271
1	3.369010	0.002457	-0.000576
1	2.157405	1.266786	1.724751
1	-0.008320	2.116334	1.404352

Table 40. $C_{10}H_{10}$ triplet twist optimized with UM06-2X:

6	0.381512	-1.427092	-0.634273
6	-1.617333	-0.898056	0.858507
6	-2.290296	0.008094	-0.000489
6	-1.606783	0.900399	-0.871478
6	-0.371215	1.419817	-0.640271
1	0.049278	-2.046306	1.419020
1	-2.144805	-1.231680	1.748575
1	-3.375248	0.018948	0.006232
1	-2.123674	1.221193	-1.772408
1	0.083025	2.016777	-1.428951
6	-0.381426	-1.426972	0.634254
6	1.617272	-0.898125	-0.858396
6	2.290202	0.007940	0.001011
6	1.606891	0.900799	0.871147
6	0.371066	1.420076	0.639936
1	-0.049076	-2.046591	-1.418968

1	2.144894	-1.231509	-1.748469
1	3.375178	0.017898	-0.004718
1	2.124042	1.222534	1.771598
1	-0.082949	2.017456	1.428405

Table 41. $C_{12}H_{12}$ singlet optimized with HF:

6	0.417282	-1.651986	0.290132
6	-0.620935	-2.215960	-0.310621
6	-2.039543	-1.872466	-0.054999
6	-2.541807	-0.678744	0.245999
6	-1.740749	0.559233	0.463608
6	-1.636163	1.593221	-0.354575
6	1.843420	-1.813486	-0.050812
6	2.713599	-0.804293	-0.024175
6	2.355261	0.608389	0.235363
6	1.248967	1.139658	-0.270856
6	0.660322	2.466142	-0.028419
6	-0.650369	2.680962	-0.117879
1	0.232472	-0.943006	1.072207
1	-0.455197	-2.946328	-1.088542
1	-2.742158	-2.679260	-0.193034
1	-3.610521	-0.599126	0.367674
1	-1.160728	0.593800	1.370648
1	-2.234198	1.644813	-1.252440
1	2.189653	-2.794099	-0.333660
1	3.752289	-1.016104	-0.221881
1	3.016540	1.186201	0.863218
1	0.666155	0.517312	-0.920161
1	1.317768	3.286386	0.209541
1	-1.027774	3.685394	-0.010164

Table 42. $C_{12}H_{12}$ singlet optimized with B3LYP:

6	0.477204	-1.624864	0.243329
6	-0.575204	-2.317071	-0.237086
6	-1.984721	-1.958363	-0.038751
6	-2.512059	-0.742446	0.221163
6	-1.736490	0.500140	0.438178
6	-1.719340	1.602934	-0.327072
6	1.888617	-1.765570	-0.070614
6	2.752689	-0.718804	-0.027969
6	2.373766	0.678112	0.197305
6	1.195473	1.178246	-0.233792
6	0.591776	2.481787	-0.010235
6	-0.741791	2.687560	-0.116908
1	0.269346	-0.797312	0.904016
1	-0.397450	-3.174453	-0.884340

1	-2.694147	-2.765717	-0.206127
1	-3.596244	-0.666213	0.269462
1	-1.075926	0.509901	1.301408
1	-2.411797	1.699494	-1.162487
1	2.264736	-2.743775	-0.359657
1	3.809680	-0.920627	-0.183889
1	3.078141	1.310360	0.735515
1	0.578924	0.512209	-0.822705
1	1.238303	3.322559	0.229519
1	-1.123079	3.703617	-0.046005

Table 43. $C_{12}H_{12}$ singlet optimized with CAM-B3LYP:

6	0.486358	-1.615805	0.258750
6	-0.544879	-2.279747	-0.273517
6	-1.961186	-1.961990	-0.042530
6	-2.496411	-0.766725	0.241652
6	-1.732575	0.484588	0.446860
6	-1.717820	1.561126	-0.339643
6	1.903059	-1.738906	-0.066010
6	2.748857	-0.693280	-0.026414
6	2.345671	0.696431	0.214583
6	1.179478	1.175837	-0.237790
6	0.561849	2.477352	-0.017841
6	-0.763300	2.664938	-0.126396
1	0.273001	-0.837145	0.975692
1	-0.351896	-3.083018	-0.981441
1	-2.654507	-2.785319	-0.191864
1	-3.577669	-0.707064	0.334441
1	-1.090846	0.521283	1.322365
1	-2.389108	1.625503	-1.193693
1	2.284002	-2.714068	-0.354970
1	3.805212	-0.876702	-0.200808
1	3.024540	1.330956	0.779922
1	0.582972	0.506677	-0.842688
1	1.202007	3.323231	0.215916
1	-1.162312	3.672758	-0.053096

Table 44. $C_{12}H_{12}$ singlet optimized with M06-2X:

6	0.502129	-1.610776	0.257796
6	-0.522724	-2.269379	-0.299752
6	-1.941849	-1.982195	-0.034695
6	-2.481398	-0.792166	0.272773
6	-1.721380	0.469659	0.451703
6	-1.730811	1.532053	-0.359045
6	1.919955	-1.716225	-0.083970
6	2.758991	-0.662990	-0.021884

6	2.330827	0.717750	0.241801
6	1.160626	1.179699	-0.227544
6	0.531278	2.480275	-0.016174
6	-0.796014	2.656624	-0.152039
1	0.286539	-0.864694	1.011181
1	-0.320991	-3.041181	-1.039829
1	-2.627328	-2.815114	-0.166894
1	-3.559071	-0.743301	0.407967
1	-1.077945	0.537975	1.326003
1	-2.406939	1.568050	-1.210864
1	2.303348	-2.682019	-0.400665
1	3.816979	-0.829684	-0.203434
1	2.989532	1.360046	0.822114
1	0.583767	0.505503	-0.849486
1	1.163165	3.330514	0.225296
1	-1.208843	3.659932	-0.095208

Table 45. $C_{12}H_{12}$ triplet optimized with UHF:

6	0.567862	-1.677868	0.272985
6	-0.540107	-2.428556	-0.220260
6	-1.910565	-2.066174	-0.048653
6	-2.454856	-0.841511	0.283552
6	-1.693103	0.425872	0.477151
6	-1.766168	1.519481	-0.318535
6	1.827593	-1.611225	-0.254774
6	2.782429	-0.542792	0.174793
6	2.453634	0.791904	0.172262
6	1.177424	1.253595	-0.255495
6	0.544475	2.490253	-0.037798
6	-0.837422	2.642640	-0.219189
1	0.366196	-1.011827	1.088070
1	-0.341357	-3.301018	-0.821661
1	-2.623499	-2.842436	-0.277835
1	-3.528362	-0.788684	0.364352
1	-1.004001	0.464735	1.300337
1	-2.543415	1.578671	-1.066352
1	2.107026	-2.237807	-1.087451
1	3.753301	-0.842612	0.532217
1	3.168642	1.496699	0.565967
1	0.568387	0.522233	-0.737795
1	1.125301	3.354093	0.245599
1	-1.240838	3.638383	-0.307205

Table 46. $C_{12}H_{12}$ triplet optimized with UB3LYP:

6	0.590801	-1.655800	0.326714
6	-0.500698	-2.480600	0.010508
6	-1.852699	-2.076561	-0.035292
6	-2.408491	-0.778441	-0.019153

6	-1.719018	0.404388	0.291422
6	-1.929403	1.718053	-0.125750
6	1.926696	-1.721349	-0.067136
6	2.778790	-0.592475	-0.005739
6	2.376829	0.755271	0.087118
6	1.081814	1.210945	-0.197584
6	0.476863	2.450033	0.058139
6	-0.905868	2.694192	-0.061333
1	0.372680	-0.782754	0.911788
1	-0.294655	-3.480153	-0.368324
1	-2.548470	-2.861188	-0.319696
1	-3.417866	-0.693579	-0.417966
1	-0.837259	0.296037	0.893789
1	-2.891552	2.012369	-0.540768
1	2.335549	-2.649947	-0.461019
1	3.848851	-0.780107	-0.045927
1	3.132043	1.481347	0.383162
1	0.449696	0.493636	-0.702619
1	1.107351	3.287569	0.351697
1	-1.213405	3.735165	-0.121750

Table 47. $C_{12}H_{12}$ triplet optimized with UCAM-B3LYP:

6	0.593118	-1.636457	0.345491
6	-0.521351	-2.486301	0.037263
6	-1.855186	-2.078842	-0.038503
6	-2.412739	-0.799252	0.008433
6	-1.700382	0.409472	0.310327
6	-1.866773	1.647920	-0.209744
6	1.851426	-1.668725	-0.152280
6	2.769193	-0.556150	0.022252
6	2.389909	0.766714	0.081619
6	1.090294	1.220023	-0.215655
6	0.487246	2.463827	0.052373
6	-0.868975	2.689097	-0.034052
1	0.398157	-0.815375	1.013083
1	-0.307300	-3.501171	-0.285721
1	-2.550841	-2.863542	-0.321259
1	-3.439850	-0.707196	-0.333157
1	-0.874585	0.319769	0.994337
1	-2.739632	1.875069	-0.818485
1	2.186901	-2.519253	-0.742272
1	3.827361	-0.786894	0.098690
1	3.143419	1.497452	0.366712
1	0.456323	0.500722	-0.711099
1	1.122559	3.299973	0.335718
1	-1.219711	3.715170	0.020209

Table 48. $C_{12}H_{12}$ triplet optimized with UM06-2X:

6	0.592402	-1.631060	0.341089

6	-0.511478	-2.476927	0.030284
6	-1.853016	-2.076847	-0.029140
6	-2.404329	-0.789564	0.002693
6	-1.695307	0.408937	0.307196
6	-1.886667	1.667538	-0.191964
6	1.873848	-1.685237	-0.133455
6	2.772578	-0.569031	0.008221
6	2.376766	0.757419	0.096046
6	1.075116	1.203700	-0.199631
6	0.478330	2.449497	0.067101
6	-0.883138	2.690097	-0.049070
1	0.401259	-0.797235	0.993015
1	-0.292740	-3.481521	-0.322457
1	-2.547767	-2.860651	-0.315819
1	-3.421813	-0.692499	-0.367193
1	-0.857461	0.325547	0.976575
1	-2.794042	1.906835	-0.742628
1	2.225210	-2.567973	-0.663644
1	3.837156	-0.781308	0.033111
1	3.122847	1.492961	0.388870
1	0.447891	0.490626	-0.717508
1	1.118492	3.278983	0.359613
1	-1.215556	3.723762	-0.047024

Table 49. $C_{14}H_{14}$ singlet optimized with HF:

6	1.476458	-1.517354	-0.304661
1	0.982567	-0.726990	-0.833557
6	2.897266	-1.269783	-0.068531
1	3.541242	-2.118723	0.093716
6	3.433388	-0.045858	-0.022837
1	4.501269	0.031363	0.104293
6	2.707729	1.232195	-0.155033
1	3.212306	2.009335	-0.710000
6	1.491774	1.464145	0.335165
1	1.041340	0.695551	0.932500
6	0.668130	2.644236	0.054926
1	1.181695	3.538464	-0.257785
6	-0.668128	2.644236	0.054925
1	-1.181692	3.538463	-0.257788
6	-1.491773	1.464145	0.335164
1	-1.041338	0.695551	0.932500
6	-2.707728	1.232197	-0.155032
1	-3.212305	2.009337	-0.709998
6	-3.433388	-0.045856	-0.022836
1	-4.501268	0.031367	0.104294
6	-2.897267	-1.269782	-0.068530
1	-3.541244	-2.118721	0.093718
6	-1.476460	-1.517354	-0.304665
1	-0.982570	-0.726994	-0.833565
6	-0.739046	-2.524343	0.156567

1	-1.212200	-3.328441	0.697275
6	0.739043	-2.524344	0.156566
1	1.212197	-3.328445	0.697271

Table 50. $C_{14}H_{14}$ singlet optimized with B3LYP:

6	-1.480216	-1.494121	0.269124
1	-0.961547	-0.662218	0.723648
6	-2.826016	-1.263473	-0.014872
1	-3.453119	-2.115856	-0.270144
6	-3.443566	-0.000024	-0.000005
1	-4.530778	0.000014	-0.000076
6	-2.825928	1.263463	0.014963
1	-3.452988	2.115823	0.270421
6	-1.480213	1.494090	-0.269117
1	-0.961539	0.662192	-0.723639
6	-0.704582	2.611083	0.039481
1	-1.200882	3.484475	0.455517
6	0.704581	2.611087	0.039483
1	1.200877	3.484477	0.455531
6	1.480217	1.494102	-0.269129
1	0.961548	0.662218	-0.723677
6	2.825928	1.263466	0.014965
1	3.452987	2.115821	0.270440
6	3.443563	-0.000025	-0.000004
1	4.530774	0.000012	-0.000073
6	2.826016	-1.263477	-0.014874
1	3.453118	-2.115857	-0.270158
6	1.480219	-1.494133	0.269131
1	0.961553	-0.662242	0.723676
6	0.704619	-2.611030	-0.039573
1	1.200883	-3.484379	-0.455743
6	-0.704621	-2.611025	-0.039570
1	-1.200892	-3.484375	-0.455731

Table 51. $C_{14}H_{14}$ singlet optimized with CAM-B3LYP:

6	1.480778	1.454366	0.304969
1	0.993413	0.644674	0.829048
6	2.751598	1.242254	-0.098583
1	3.307129	2.070340	-0.534858
6	3.441880	-0.029663	-0.017451
1	4.524287	0.019169	0.058846
6	2.873753	-1.261594	-0.050174
1	3.513861	-2.124107	0.114708
6	1.475703	-1.493735	-0.285068
1	0.968110	-0.673297	-0.774895
6	0.726502	-2.540235	0.118956

1	1.211461	-3.374328	0.618723
6	-0.726502	-2.540235	0.118956
1	-1.211462	-3.374328	0.618723
6	-1.475703	-1.493735	-0.285069
1	-0.968110	-0.673297	-0.774896
6	-2.873753	-1.261594	-0.050174
1	-3.513862	-2.124106	0.114708
6	-3.441880	-0.029663	-0.017451
1	-4.524287	0.019170	0.058846
6	-2.751597	1.242254	-0.098583
1	-3.307129	2.070341	-0.534858
6	-1.480777	1.454366	0.304968
1	-0.993412	0.644674	0.829047
6	-0.678292	2.616864	0.028512
1	-1.193240	3.508010	-0.318530
6	0.678293	2.616864	0.028512
1	1.193241	3.508010	-0.318530

Table 52. $C_{14}H_{14}$ singlet optimized with M06-2X:

6	1.481684	1.439705	0.312966
1	1.001635	0.627520	0.844335
6	2.756785	1.240259	-0.097887
1	3.301459	2.075209	-0.535332
6	3.454040	-0.028800	-0.013722
1	4.536053	0.018238	0.069287
6	2.876135	-1.259973	-0.047488
1	3.504124	-2.129011	0.131733
6	1.477043	-1.479721	-0.299883
1	0.978004	-0.663605	-0.809493
6	0.726813	-2.524618	0.119145
1	1.215521	-3.347344	0.634496
6	-0.726814	-2.524618	0.119147
1	-1.215520	-3.347342	0.634504
6	-1.477045	-1.479724	-0.299885
1	-0.978007	-0.663613	-0.809503
6	-2.876136	-1.259973	-0.047487
1	-3.504126	-2.129010	0.131738
6	-3.454039	-0.028799	-0.013722
1	-4.536051	0.018240	0.069288
6	-2.756784	1.240260	-0.097888
1	-3.301456	2.075209	-0.535339
6	-1.481685	1.439709	0.312968
1	-1.001638	0.627528	0.844345
6	-0.680111	2.601841	0.026561
1	-1.197834	3.486826	-0.333185
6	0.680113	2.601840	0.026561
1	1.197837	3.486824	-0.333184

Table 53. TS $C_{14}H_{14}$ optimized with CAM-B3LYP:

6	1.472548	-1.474810	-0.273507
1	0.959377	-0.643927	-0.733822
6	2.813745	-1.254805	0.013708
1	3.433100	-2.111722	0.268609
6	3.433616	-0.000002	0.000002
1	4.519786	0.000002	0.000014
6	2.813735	1.254803	-0.013720
1	3.433084	2.111718	-0.268648
6	1.472547	1.474806	0.273505
1	0.959376	0.643923	0.733818
6	0.700957	2.586310	-0.040191
1	1.199119	3.454449	-0.462706
6	-0.700957	2.586309	-0.040191
1	-1.199121	3.454448	-0.462706
6	-1.472547	1.474805	0.273506
1	-0.959376	0.643922	0.733819
6	-2.813735	1.254803	-0.013720
1	-3.433084	2.111717	-0.268648
6	-3.433616	-0.000002	0.000002
1	-4.519786	0.000001	0.000013
6	-2.813744	-1.254805	0.013708
1	-3.433100	-2.111722	0.268608
6	-1.472547	-1.474809	-0.273507
1	-0.959376	-0.643926	-0.733821
6	-0.700960	-2.586303	0.040203
1	-1.199121	-3.454435	0.462736
6	0.700960	-2.586303	0.040204
1	1.199120	-3.454435	0.462737

Table 54. TS $C_{14}H_{14}$ optimized with M06-2X:

6	1.474615	-1.459049	-0.288679
1	0.973097	-0.631214	-0.771842
6	2.817288	-1.252185	0.017098
1	3.422150	-2.115517	0.285765
6	3.447295	0.000001	0.000005
1	4.533407	0.000001	0.000017
6	2.817289	1.252186	-0.017102
1	3.422147	2.115516	-0.285780
6	1.474615	1.459048	0.288675
1	0.973096	0.631211	0.771834

6	0.702060	2.568641	-0.040733
1	1.202578	3.426056	-0.482630
6	-0.702061	2.568641	-0.040733
1	-1.202579	3.426055	-0.482630
6	-1.474615	1.459047	0.288674
1	-0.973096	0.631210	0.771832
6	-2.817289	1.252185	-0.017102
1	-3.422147	2.115516	-0.285779
6	-3.447295	0.000001	0.000005
1	-4.533408	0.000000	0.000016
6	-2.817287	-1.252185	0.017098
1	-3.422149	-2.115518	0.285764
6	-1.474614	-1.459048	-0.288677
1	-0.973095	-0.631212	-0.771838
6	-0.702059	-2.568641	0.040735
1	-1.202577	-3.426051	0.482640
6	0.702060	-2.568641	0.040735
1	1.202577	-3.426051	0.482641

Table 55. $C_{14}H_{14}$ triplet optimized with UHF:

6	-1.437809	-1.522156	0.209391
1	-0.906104	-0.723193	0.676282
6	-2.768825	-1.271865	-0.105910
1	-3.387217	-2.093373	-0.432984
6	-3.397672	0.000803	-0.000263
1	-4.475211	0.001162	-0.000640
6	-2.768113	1.272975	0.105518
1	-3.386386	2.095122	0.431192
6	-1.436456	1.522358	-0.208394
1	-0.904557	0.722724	-0.673907
6	-0.679630	2.718310	0.038892
1	-1.207067	3.609703	0.338268
6	0.674439	2.757953	-0.041576
1	1.190123	3.679777	0.168116
6	1.494227	1.548360	-0.333215
1	1.051887	0.809877	-0.972468
6	2.723549	1.262556	0.177056
1	3.263568	2.032029	0.708465
6	3.388511	-0.000853	-0.000266
1	4.466068	-0.000886	-0.001051
6	2.723285	-1.263827	-0.176541
1	3.263639	-2.034408	-0.705986
6	1.492876	-1.548472	0.332654
1	1.050220	-0.809011	0.970491
6	0.672846	-2.757924	0.041296
1	1.188430	-3.679714	-0.168826
6	-0.681015	-2.718264	-0.038492

1	-1.208669	-3.609535	-0.337851

Table 56. $C_{14}H_{14}$ triplet optimized with UB3LYP:

6	-1.418957	1.494175	-0.217200
1	-0.882417	0.639069	-0.600813
6	-2.791962	1.280860	0.042203
1	-3.407175	2.148931	0.271019
6	-3.414369	0.050107	0.031571
1	-4.495567	0.034230	0.139661
6	-2.756890	-1.243882	-0.145026
1	-3.300601	-1.992712	-0.720271
6	-1.524515	-1.542600	0.328024
1	-1.058072	-0.795250	0.959542
6	-0.715315	-2.704335	0.012699
1	-1.219109	-3.611245	-0.308943
6	0.666092	-2.660738	-0.030384
1	1.192729	-3.551519	-0.365185
6	1.418956	-1.494175	0.217200
1	0.882416	-0.639070	0.600814
6	2.791961	-1.280860	-0.042203
1	3.407175	-2.148931	-0.271018
6	3.414369	-0.050107	-0.031571
1	4.495567	-0.034230	-0.139660
6	2.756890	1.243882	0.145026
1	3.300601	1.992712	0.720271
6	1.524516	1.542600	-0.328024
1	1.058073	0.795250	-0.959543
6	0.715315	2.704335	-0.012700
1	1.219109	3.611245	0.308942
6	-0.666092	2.660738	0.030383
1	-1.192728	3.551519	0.365184

Table 57. $C_{14}H_{14}$ triplet optimized with UCAM-B3LYP:

6	1.539914	-1.537006	-0.346495
1	1.092331	-0.816823	-1.021442
6	2.736553	-1.217966	0.169015
1	3.252909	-1.936566	0.803007
6	3.392864	0.076167	-0.031246
1	4.471599	0.063616	-0.152880
6	2.762047	1.290450	-0.046779
1	3.364448	2.166304	-0.275191
6	1.386213	1.487848	0.208214
1	0.851254	0.627473	0.579892
6	0.639520	2.655159	-0.031398
1	1.169391	3.545179	-0.360169
6	-0.732934	2.702030	0.021308

1	-1.241023	3.608693	-0.290403
6	-1.539919	1.537009	0.346501
1	-1.092345	0.816829	1.021457
6	-2.736551	1.217967	-0.169023
1	-3.252898	1.936565	-0.803024
6	-3.392863	-0.076167	0.031231
1	-4.471600	-0.063615	0.152854
6	-2.762047	-1.290451	0.046771
1	-3.364451	-2.166303	0.275181
6	-1.386211	-1.487851	-0.208210
1	-0.851249	-0.627478	-0.579890
6	-0.639520	-2.655161	0.031409
1	-1.169391	-3.545180	0.360181
6	0.732934	-2.702028	-0.021298
1	1.241025	-3.608691	0.290415

Table 58. $C_{14}H_{14}$ triplet optimized with UM06-2X:

6	-1.341137	1.487626	-0.204526
1	-0.823496	0.617757	-0.583803
6	-2.725554	1.330973	0.049681
1	-3.297371	2.227316	0.277980
6	-3.397069	0.137238	0.025220
1	-4.476684	0.156742	0.135706
6	-2.768803	-1.170682	-0.17334
1	-3.289458	-1.882636	-0.811510
6	-1.578940	-1.506126	0.356671
1	-1.129569	-0.793893	1.041726
6	-0.790934	-2.684365	0.025804
1	-1.314217	-3.582810	-0.284751
6	0.581453	-2.651220	-0.033493
1	1.105044	-3.543847	-0.365535
6	1.341140	-1.487621	0.204527
1	0.823504	-0.617743	0.583792
6	2.725556	-1.330973	-0.049687
1	3.297371	-2.227317	-0.277984
6	3.397071	-0.137238	-0.025237
1	4.476685	-0.156741	-0.135735
6	2.768804	1.170681	0.17333
1	3.28947	1.882638	0.811487
6	1.578932	1.506120	-0.356663
1	1.129546	0.793880	-1.041703
6	0.790934	2.684363	-0.025792
1	1.314221	3.582808	0.28476
6	-0.581452	2.651223	0.033508
1	-1.105042	3.543849	0.365553

Table 59. $C_{16}H_{16}$ S_4 singlet optimized with HF:

6	-0.000688	2.372164	0.046538
6	0.000688	-2.372164	0.046538
6	-2.372164	-0.000688	-0.046538
6	2.372164	0.000688	-0.046538
6	-1.204276	2.912613	-0.108855
6	1.204276	-2.912613	-0.108855
6	-2.912613	-1.204276	0.108855
6	2.912613	1.204276	0.108855
6	-2.458735	2.442074	0.504684
6	2.458735	-2.442074	0.504684
6	-2.442074	-2.458735	-0.504684
6	2.442074	2.458735	-0.504684
6	-2.942909	1.204276	0.558062
6	2.942909	-1.204276	0.558062
6	-1.204276	-2.942909	-0.558062
6	1.204276	2.942909	-0.558062
1	0.117108	1.505960	0.671751
1	-0.117108	-1.505960	0.671751
1	-1.505960	0.117108	-0.671751
1	1.505960	-0.117108	-0.671751
1	-1.291686	3.816885	-0.693298
1	1.291686	-3.816885	-0.693298
1	-3.816885	-1.291686	0.693298
1	3.816885	1.291686	0.693298
1	-3.062456	3.222309	0.941550
1	3.062456	-3.222309	0.941550
1	-3.222309	-3.062456	-0.941550
1	3.222309	3.062456	-0.941550
1	-3.879930	1.062610	1.072132
1	3.879930	-1.062610	1.072132
1	-1.062610	-3.879930	-1.072132
1	1.062610	3.879930	-1.072132

Table 60. $C_{16}H_{16}$ S_4 singlet optimized with B3LYP:

6	0.006911	2.365967	0.028014
6	-0.006911	-2.365967	0.028014
6	-2.365967	0.006911	-0.028014
6	2.365967	-0.006911	-0.028014
6	-1.195834	2.968721	-0.120359
6	1.195834	-2.968721	-0.120359
6	-2.968721	-1.195834	0.120359
6	2.968721	1.195834	0.120359
6	-2.500065	2.500065	0.318535
6	2.500065	-2.500065	0.318535
6	-2.500065	-2.500065	-0.318535
6	2.500065	2.500065	-0.318535
6	-2.988269	1.238664	0.399992

6	2.988269	-1.238664	0.399992
6	-1.238664	-2.988269	-0.399992
6	1.238664	2.988269	-0.399992
1	0.070275	1.416151	0.546210
1	-0.070275	-1.416151	0.546210
1	-1.416151	0.070275	-0.546210
1	1.416151	-0.070275	-0.546210
1	-1.198004	3.960091	-0.572901
1	1.198004	-3.960091	-0.572901
1	-3.960091	-1.198004	0.572901
1	3.960091	1.198004	0.572901
1	-3.189060	3.296414	0.591208
1	3.189060	-3.296414	0.591208
1	-3.296414	-3.189060	-0.591208
1	3.296414	3.189060	-0.591208
1	-3.998023	1.132409	0.790844
1	3.998023	-1.132409	0.790844
1	-1.132409	-3.998023	-0.790844
1	1.132409	3.998023	-0.790844

Table 61. $C_{16}H_{16}$ S_4 singlet optimized with CAM-B3LYP:

6	-0.002499	2.349328	0.025049
6	0.002499	-2.349328	0.025049
6	-2.349328	-0.002499	-0.025049
6	2.349328	0.002499	-0.025049
6	-1.205495	2.924731	-0.111121
6	1.205495	-2.924731	-0.111121
6	-2.924731	-1.205495	0.111121
6	2.924731	1.205495	0.111121
6	-2.474403	2.458611	0.437036
6	2.474403	-2.458611	0.437036
6	-2.458611	-2.474403	-0.437036
6	2.458611	2.474403	-0.437036
6	-2.948620	1.205495	0.509360
6	2.948620	-1.205495	0.509360
6	-1.205495	-2.948620	-0.509360
6	1.205495	2.948620	-0.509360
1	0.095834	1.439856	0.606276
1	-0.095834	-1.439856	0.606276
1	-1.439856	0.095834	-0.606276
1	1.439856	-0.095834	-0.606276
1	-1.251245	3.877033	-0.637091
1	1.251245	-3.877033	-0.637091
1	-3.877033	-1.251245	0.637091
1	3.877033	1.251245	0.637091
1	-3.124585	3.248317	0.804458
1	3.124585	-3.248317	0.804458

1	-3.248317	-3.124585	-0.804458
1	3.248317	3.124585	-0.804458
1	-3.919988	1.069717	0.977682
1	3.919988	-1.069717	0.977682
1	-1.069717	-3.919988	-0.977682
1	1.069717	3.919988	-0.977682

Table 62. $C_{16}H_{16}$ S_4 singlet optimized with M06-2X:

6	0.000259	2.328381	0.010056
6	-0.000259	-2.328381	0.010056
6	-2.328381	0.000259	-0.010056
6	2.328381	-0.000259	-0.010056
6	-1.203517	2.910973	-0.119383
6	1.203517	-2.910973	-0.119383
6	-2.910973	-1.203517	0.119383
6	2.910973	1.203517	0.119383
6	-2.456612	2.456612	0.477467
6	2.456612	-2.456612	0.477467
6	-2.456612	-2.456612	-0.477467
6	2.456612	2.456612	-0.477467
6	-2.919065	1.197387	0.563943
6	2.919065	-1.197387	0.563943
6	-1.197387	-2.919065	-0.563943
6	1.197387	2.919065	-0.563943
1	0.105024	1.434937	0.618737
1	-0.105024	-1.434937	0.618737
1	-1.434937	0.105024	-0.618737
1	1.434937	-0.105024	-0.618737
1	-1.256262	3.849431	-0.669337
1	1.256262	-3.849431	-0.669337
1	-3.849431	-1.256262	0.669337
1	3.849431	1.256262	0.669337
1	-3.093928	3.245882	0.867544
1	3.093928	-3.245882	0.867544
1	-3.245882	-3.093928	-0.867544
1	3.245882	3.093928	-0.867544
1	-3.871401	1.047513	1.066440
1	3.871401	-1.047513	1.066440
1	-1.047513	-3.871401	-1.066440
1	1.047513	3.871401	-1.066440

Table 63. $C_{16}H_{16}$ C_1 singlet optimized with HF:

6	0.016383	2.256423	0.042598
6	1.330191	2.895499	-0.028018
6	-1.174733	2.848428	0.036999
6	2.475057	2.215747	-0.113669

6	-2.432404	2.085373	0.167594
6	2.547335	0.756832	-0.283467
6	-2.568417	0.834725	-0.260390
6	3.548379	-0.033379	0.089805
6	-3.708117	-0.055089	-0.014178
6	3.558237	-1.499510	-0.084032
6	-3.645500	-1.386463	-0.047643
6	2.523539	-2.334042	0.017029
6	-2.420938	-2.183409	-0.269744
6	1.162811	-1.951459	0.410545
6	-1.262456	-1.930871	0.329693
6	0.034045	-2.529099	0.007999
1	0.029143	1.190639	0.162619
1	1.364376	3.969791	0.046637
1	-1.247289	3.923510	-0.017228
1	3.406997	2.754191	-0.065832
1	-3.242975	2.570627	0.689262
1	1.706662	0.298783	-0.768681
1	-1.758521	0.399216	-0.815115
1	4.435289	0.399563	0.527473
1	-4.656348	0.408306	0.205362
1	4.517300	-1.926692	-0.329940
1	-4.561030	-1.940859	0.084424
1	2.689696	-3.374361	-0.210659
1	-2.492729	-2.995978	-0.977433
1	1.087396	-1.085618	1.046192
1	-1.232696	-1.147969	1.069074
1	0.054254	-3.381378	-0.652861

Table 64. $C_{16}H_{16}$ C_1 singlet optimized with B3LYP:

6	0.001441	2.277533	-0.034996
6	1.299313	2.905070	-0.037475
6	-1.215405	2.866845	0.037889
6	2.470431	2.211159	-0.064425
6	-2.461518	2.111486	0.128053
6	2.549289	0.773959	-0.223612
6	-2.574510	0.807994	-0.215776
6	3.600674	-0.028946	0.061021
6	-3.706691	-0.066346	-0.006168
6	3.599185	-1.480763	-0.084082
6	-3.650589	-1.422724	-0.051695
6	2.536443	-2.322033	-0.002503
6	-2.443978	-2.229350	-0.220379
6	1.191393	-1.939661	0.377481
6	-1.241648	-1.930028	0.321676
6	0.033345	-2.539941	0.014078
1	0.011521	1.194910	-0.022340

1	1.336190	3.987911	0.048031
1	-1.282867	3.950785	0.097992
1	3.403345	2.756095	0.053443
1	-3.318883	2.628309	0.553852
1	1.670148	0.294477	-0.634191
1	-1.717052	0.336646	-0.682636
1	4.533486	0.422124	0.395694
1	-4.669977	0.399365	0.188405
1	4.567462	-1.929203	-0.292870
1	-4.589137	-1.967825	0.016279
1	2.703416	-3.369692	-0.241116
1	-2.535146	-3.119607	-0.841508
1	1.111362	-1.038139	0.979949
1	-1.193952	-1.090097	1.010023
1	0.047036	-3.421593	-0.623533

Table 65. $C_{16}H_{16}$ C_1 singlet optimized with CAM-B3LYP:

6	0.003504	2.247989	-0.004428
6	1.303370	2.884897	-0.040807
6	-1.198472	2.841888	0.045247
6	2.463481	2.202516	-0.093814
6	-2.446775	2.085971	0.154184
6	2.541211	0.758645	-0.251032
6	-2.568918	0.809145	-0.235033
6	3.577394	-0.029642	0.070177
6	-3.703450	-0.068445	-0.011284
6	3.583767	-1.484173	-0.077093
6	-3.644143	-1.411958	-0.052702
6	2.528812	-2.313808	0.016079
6	-2.426951	-2.202631	-0.241663
6	1.181535	-1.917770	0.391519
6	-1.245914	-1.902968	0.317348
6	0.037227	-2.506194	0.011319
1	0.014422	1.167008	0.052052
1	1.332270	3.967648	0.036451
1	-1.262756	3.926778	0.062630
1	3.399241	2.746938	-0.009899
1	-3.286381	2.585401	0.631086
1	1.675540	0.280714	-0.690322
1	-1.730553	0.354379	-0.749765
1	4.492727	0.424506	0.443564
1	-4.662071	0.400030	0.193464
1	4.550348	-1.929519	-0.294542
1	-4.573552	-1.966311	0.042490
1	2.687576	-3.365440	-0.205112
1	-2.501020	-3.072373	-0.891462
1	1.105743	-1.023492	1.004061

1	-1.210618	-1.074699	1.019516
1	0.055018	-3.382336	-0.632309

Table 66. $C_{16}H_{16}$ C_1 singlet optimized with M06-2X:

6	0.024008	2.208596	-0.003156
6	1.321877	2.856048	-0.046287
6	-1.171587	2.822297	0.051168
6	2.487453	2.179944	-0.105956
6	-2.429162	2.081726	0.170741
6	2.562964	0.734477	-0.275354
6	-2.572049	0.815485	-0.253670
6	3.588567	-0.054355	0.087191
6	-3.717743	-0.048525	-0.012591
6	3.584533	-1.510226	-0.065564
6	-3.669003	-1.395079	-0.050335
6	2.506253	-2.314503	0.018559
6	-2.446830	-2.180286	-0.239626
6	1.167009	-1.881605	0.392317
6	-1.265561	-1.855273	0.313457
6	0.016570	-2.464285	0.010490
1	0.029207	1.125069	0.063803
1	1.339873	3.938867	0.037273
1	-1.215392	3.908552	0.066984
1	3.423199	2.724003	-0.016899
1	-3.252400	2.573915	0.682888
1	1.715140	0.260128	-0.757032
1	-1.753656	0.366399	-0.808092
1	4.488138	0.398198	0.499211
1	-4.667586	0.433022	0.203528
1	4.544600	-1.972822	-0.275545
1	-4.598376	-1.947353	0.055165
1	2.634370	-3.370220	-0.204429
1	-2.511603	-3.063082	-0.872602
1	1.104172	-0.982075	1.001654
1	-1.234471	-1.014161	1.003503
1	0.030995	-3.345056	-0.627712

Table 67. $C_{16}H_{16}$ triplet optimized with UHF:

6	0.000001	2.329827	-0.100504
6	1.256510	2.913567	-0.004274
6	-1.256567	2.913470	-0.004431
6	2.436565	2.156083	0.039670
6	-2.436748	2.156092	0.039586
6	2.509769	0.786553	-0.182550
6	-2.509983	0.786493	-0.182170
6	3.608304	-0.040525	0.032617

6	-3.608497	-0.040637	0.032431
6	3.591475	-1.435273	-0.049847
6	-3.591340	-1.435462	-0.049900
6	2.485566	-2.294467	-0.092644
6	-2.485410	-2.294612	-0.092365
6	1.195135	-1.950907	0.296694
6	-1.194916	-1.951003	0.296920
6	0.000098	-2.602114	0.008026
1	0.000118	1.263250	-0.181965
1	1.332081	3.978478	0.134901
1	-1.332131	3.978421	0.134459
1	3.343769	2.673227	0.309639
1	-3.343924	2.673454	0.309200
1	1.635261	0.309341	-0.569761
1	-1.635253	0.309232	-0.568744
1	4.559554	0.422659	0.242447
1	-4.559947	0.422346	0.241754
1	4.557012	-1.910001	-0.119992
1	-4.556812	-1.910294	-0.120300
1	2.660076	-3.298752	-0.443760
1	-2.659891	-3.298934	-0.443387
1	1.095002	-1.040694	0.859219
1	-1.094733	-1.040871	0.859554
1	0.000048	-3.513374	-0.566818

Table 68. $C_{16}H_{16}$ triplet optimized with UB3LYP:

6	-0.000480	2.313217	-0.106583
6	1.259852	2.906144	0.006812
6	-1.260948	2.905919	0.006758
6	2.461739	2.180113	0.032564
6	-2.462646	2.179574	0.032616
6	2.542688	0.791789	-0.167499
6	-2.543089	0.791243	-0.167296
6	3.637362	-0.040106	0.016291
6	-3.637467	-0.041178	0.016171
6	3.613928	-1.453605	-0.089518
6	-3.613306	-1.454632	-0.089375
6	2.501446	-2.300923	-0.086627
6	-2.500555	-2.301568	-0.086632
6	1.208148	-1.947713	0.326798
6	-1.207189	-1.947910	0.326463
6	0.000495	-2.584155	0.032122
1	-0.000416	1.237524	-0.219003
1	1.314658	3.981218	0.157321
1	-1.315976	3.981020	0.156993
1	3.373662	2.719975	0.274889
1	-3.374698	2.719236	0.274935
1	1.642654	0.305979	-0.516410
1	-1.642754	0.305763	-0.515908
1	4.603210	0.419629	0.217426
1	-4.603643	0.418118	0.216696
1	4.580861	-1.933417	-0.217435

1	-4.580063	-1.934906	-0.216949
1	2.659989	-3.319842	-0.434654
1	-2.658803	-3.320620	-0.434394
1	1.118521	-1.028720	0.897418
1	-1.117780	-1.028704	0.896776
1	0.000724	-3.499512	-0.556089

Table 69. $C_{16}H_{16}$ triplet optimized with UCAM-B3LYP:

6	-0.000253	2.259726	-0.129711
6	1.254332	2.868412	0.025538
6	-1.254860	2.868318	0.025574
6	2.450892	2.184370	0.022780
6	-2.451331	2.184126	0.022828
6	2.556996	0.775321	-0.229381
6	-2.557167	0.775086	-0.229419
6	3.597679	-0.031741	0.068165
6	-3.597659	-0.032222	0.068146
6	3.591527	-1.472041	-0.126704
6	-3.591216	-1.472530	-0.126722
6	2.504077	-2.292087	-0.074339
6	-2.503645	-2.292419	-0.074346
6	1.203946	-1.903272	0.350442
6	-1.203552	-1.903459	0.350470
6	0.000233	-2.521297	0.046040
1	-0.000230	1.192873	-0.301644
1	1.278406	3.935184	0.230160
1	-1.279041	3.935080	0.230242
1	3.357518	2.724164	0.277823
1	-3.358026	2.723757	0.277971
1	1.703331	0.307196	-0.700596
1	-1.703411	0.307181	-0.700686
1	4.512477	0.408427	0.459436
1	-4.512538	0.407745	0.459453
1	4.551526	-1.927847	-0.349638
1	-4.551140	-1.928502	-0.349638
1	2.640493	-3.326262	-0.380805
1	-2.639917	-3.326610	-0.380817
1	1.136859	-0.981487	0.918395
1	-1.136592	-0.981721	0.918506
1	0.000298	-3.434925	-0.544333

Table 70. $C_{16}H_{16}$ triplet optimized with UM06-2X:

6	-0.000184	2.225312	-0.109870
6	1.250715	2.849853	0.015868
6	-1.251260	2.849810	0.015738
6	2.454454	2.170110	0.022030
6	-2.454831	2.169892	0.021604
6	2.561427	0.766093	-0.229549
6	-2.561505	0.765760	-0.229932

6	3.616902	-0.036383	0.067574
6	-3.616797	-0.036822	0.067642
6	3.606773	-1.471267	-0.111809
6	-3.606591	-1.471642	-0.111477
6	2.499189	-2.277166	-0.079065
6	-2.498820	-2.277445	-0.078779
6	1.206050	-1.871330	0.345901
6	-1.205709	-1.871374	0.345818
6	0.000210	-2.486587	0.034683
1	-0.000195	1.147994	-0.220692
1	1.265036	3.921862	0.192746
1	-1.265662	3.921805	0.192696
1	3.359084	2.712493	0.280010
1	-3.359694	2.712044	0.279255
1	1.712665	0.292626	-0.707444
1	-1.712782	0.292386	-0.708021
1	4.534432	0.415957	0.438244
1	-4.534245	0.415569	0.438487
1	4.565756	-1.943176	-0.302881
1	-4.565528	-1.943741	-0.302327
1	2.617701	-3.311642	-0.392717
1	-2.617292	-3.312031	-0.392093
1	1.144621	-0.955250	0.927224
1	-1.144288	-0.955107	0.926860
1	0.000261	-3.392677	-0.567617

Table 71. $C_{18}H_{18}$ singlet optimized with HF:

6	0.027472	-2.980306	0.212872
6	1.209013	-3.522172	-0.072460
1	1.262054	-4.540568	-0.424964
6	2.482085	-2.787083	0.033903
1	3.370168	-3.366202	0.233509
6	2.569237	-1.469262	-0.130873
1	1.668727	-0.949338	-0.397080
6	3.774002	-0.646923	-0.030336
1	4.720987	-1.160758	-0.033737
1	4.707966	1.212094	0.033981
6	3.766680	0.687895	0.030182
6	2.552937	1.496960	0.130342
1	1.657959	0.967023	0.395484
1	3.333403	3.402786	-0.232125
6	2.451563	2.813874	-0.033472
6	1.170544	3.535055	0.072817
1	1.212503	4.553768	0.425913
1	0.006870	1.986470	-0.617376
6	-0.004973	2.980558	-0.213041
6	-1.318865	3.578892	0.013592
1	-1.340273	4.632682	0.237388
6	-2.471787	2.902888	0.023899
1	-3.378487	3.438497	0.251849

6	-2.593013	1.462325	-0.187706
1	-1.722317	0.955830	-0.558001
6	-3.665128	0.714310	0.062589
1	-4.587262	1.178466	0.376476
6	-3.657089	-0.754124	-0.062562
1	-4.574007	-1.228327	-0.376720
6	-2.576945	-1.490431	0.187932
1	-1.711895	-0.974553	0.558513
6	-2.440017	-2.929545	-0.023930
1	-3.340816	-3.474967	-0.251996
1	-1.289693	-4.646845	-0.237752
6	-1.279779	-3.592925	-0.013766
1	0.028487	-1.985972	0.616745

Table 72. $C_{18}H_{18}$ singlet optimized with B3LYP:

6	-1.539947	2.550712	-0.000116
6	-0.776547	3.714831	0.000015
1	-1.295799	4.669903	0.000126
6	0.632118	3.742026	-0.000018
1	1.114154	4.716409	-0.000134
6	1.439941	2.608291	0.000117
1	0.917289	1.660305	0.000426
6	2.829969	2.530226	-0.000034
1	3.396727	3.457906	-0.000179
1	4.643404	1.394406	0.000022
6	3.558580	1.324268	-0.000006
6	2.980686	0.057726	-0.000055
1	1.898465	0.036777	-0.000221
1	4.693918	-1.213539	0.000086
6	3.607192	-1.185505	0.000022
6	2.925903	-2.418822	0.000007
1	3.528209	-3.323815	-0.000038
1	0.981032	-1.623643	0.000210
6	1.539947	-2.550712	0.000064
6	0.776545	-3.714833	-0.000004
1	1.295798	-4.669904	-0.000054
6	-0.632116	-3.742028	0.000002
1	-1.114153	-4.716410	0.000056
6	-1.439942	-2.608290	-0.000068
1	-0.917289	-1.660305	-0.000219
6	-2.829966	-2.530227	-0.000004
1	-3.396725	-3.457906	0.000051
6	-3.558578	-1.324266	-0.000016
1	-4.643403	-1.394405	-0.000062
6	-2.980686	-0.057726	0.000045
1	-1.898465	-0.036777	0.000184
6	-3.607193	1.185508	0.000011

1	-4.693919	1.213540	-0.000002
1	-3.528211	3.323815	0.000184
6	-2.925907	2.418822	0.000036
1	-0.981033	1.623643	-0.000419

Table 73. $C_{18}H_{18}$ singlet (C_1) optimized with CAM-B3LYP:

6	0.015783	2.962845	-0.129637
6	1.223711	3.542681	0.019649
1	1.278408	4.611633	0.207485
6	2.479706	2.815970	-0.038072
1	3.388516	3.402078	-0.144462
6	2.568913	1.474234	0.059009
1	1.644617	0.938860	0.232694
6	3.754678	0.665936	0.001945
1	4.709774	1.182362	-0.019068
1	4.701736	-1.213816	0.019186
6	3.750126	-0.690996	-0.001868
6	2.558970	-1.491324	-0.058995
1	1.638298	-0.949758	-0.232726
1	3.365628	-3.424611	0.144480
6	2.460765	-2.832434	0.038067
6	1.199938	-3.550739	-0.019711
1	1.247509	-4.620028	-0.207575
1	-0.002951	-1.908621	0.374112
6	-0.004109	-2.962886	0.129592
6	-1.294839	-3.571135	-0.029762
1	-1.319937	-4.643855	-0.197383
6	-2.468084	-2.889462	-0.039142
1	-3.385536	-3.443833	-0.213404
6	-2.581781	-1.466072	0.114135
1	-1.671265	-0.939247	0.369149
6	-3.689499	-0.712715	-0.037727
1	-4.643061	-1.199841	-0.223578
6	-3.684633	0.737338	0.037815
1	-4.634900	1.230850	0.223695
6	-2.571894	1.483246	-0.114110
1	-1.664952	0.950312	-0.369161
6	-2.448650	2.905848	0.039119
1	-3.362367	3.466352	0.213379
1	-1.288821	4.652541	0.197281
6	-1.270875	3.579672	0.029695
1	0.009945	1.908583	-0.374100

Table 74. $C_{18}H_{18}$ singlet (C_1) optimized with M06-2X:

6	-0.000344	2.955691	0.149552
6	-1.203352	3.547715	-0.020754

1	-1.244559	4.612546	-0.235163
6	-2.464259	2.828109	0.051610
1	-3.370654	3.411814	0.189902
6	-2.556407	1.485525	-0.073863
1	-1.641070	0.950822	-0.297716
6	-3.752731	0.688615	-0.001520
1	-4.702184	1.215498	0.026086
1	-4.708016	-1.192831	-0.026072
6	-3.756023	-0.670552	0.001554
6	-2.563574	-1.473247	0.073886
1	-1.645648	-0.942986	0.297717
1	-3.387134	-3.395572	-0.189940
6	-2.477934	-2.816258	-0.051610
6	-1.220511	-3.541928	0.020718
1	-1.266829	-4.606562	0.235064
1	-0.014972	-1.910650	-0.434595
6	-0.014690	-2.955655	-0.149557
6	1.270889	-3.573081	0.037361
1	1.282192	-4.641745	0.231848
6	2.451112	-2.898272	0.048855
1	3.363568	-3.451864	0.251070
6	2.573638	-1.475928	-0.128539
1	1.677381	-0.942608	-0.421567
6	3.690302	-0.734311	0.044497
1	4.633414	-1.231775	0.255906
6	3.693823	0.716548	-0.044458
1	4.639331	1.209427	-0.255906
6	2.580772	1.463574	0.128573
1	1.681939	0.934621	0.421626
6	2.465125	2.886485	-0.048876
1	3.380236	3.435666	-0.251121
1	1.304587	4.635547	-0.231936
6	1.288165	3.566958	-0.037404
1	-0.005592	1.910717	0.434655

Table 75. $C_{18}H_{18}$ TS optimized with CAM-B3LYP:

6	1.746946	-2.391187	-0.000097
6	1.094539	-3.614818	0.000014
1	1.697786	-4.518011	0.000108
6	-0.299789	-3.764908	-0.000017
1	-0.696994	-4.775795	-0.000117
6	-1.197701	-2.708181	0.000098
1	-0.760750	-1.719667	0.000369
6	-2.583589	-2.755327	-0.000031
1	-3.063929	-3.729465	-0.000151
1	-4.484950	-1.784639	0.000029
6	-3.410915	-1.623033	-0.000006

6	-2.944733	-0.317059	-0.000061
1	-1.870156	-0.201316	-0.000227
1	-4.762140	0.788860	0.000079
6	-3.678326	0.859681	0.000016
6	-3.111088	2.142186	0.000002
1	-3.787844	2.991699	-0.000040
1	-1.109572	1.518357	0.000197
6	-1.746946	2.391188	0.000057
6	-1.094536	3.614825	-0.000005
1	-1.697784	4.518017	-0.000054
6	0.299785	3.764913	0.000004
1	0.696991	4.775800	0.000052
6	1.197701	2.708181	-0.000058
1	0.760749	1.719667	-0.000197
6	2.583582	2.755326	0.000000
1	3.063924	3.729464	0.000046
6	3.410913	1.623026	-0.000012
1	4.484947	1.784633	-0.000064
6	2.944732	0.317059	0.000054
1	1.870156	0.201315	0.000201
6	3.678330	-0.859687	0.000009
1	4.762144	-0.788864	-0.000016
1	3.787850	-2.991699	0.000152
6	3.111095	-2.142185	0.000032
1	1.109573	-1.518356	-0.000361

Table 76. $C_{18}H_{18}$ TS optimized with M06-2X:

6	-1.479250	2.562445	0.018412
6	-0.702569	3.714763	-0.004560
1	-1.209971	4.675168	-0.024924
6	0.702561	3.714767	0.003297
1	1.209960	4.675180	0.023330
6	1.479246	2.562442	-0.019281
1	0.939358	1.627400	-0.074465
6	2.865496	2.465745	0.004778
1	3.443691	3.385273	0.025081
1	4.653265	1.289137	-0.020337
6	3.567775	1.248749	-0.001714
6	2.958007	0.000007	0.020786
1	1.878184	0.000015	0.074059
1	4.653267	-1.289134	-0.019895
6	3.567777	-1.248742	-0.001287
6	2.865501	-2.465737	0.005619
1	3.443696	-3.385257	0.026240
1	0.939361	-1.627422	-0.073920
6	1.479250	-2.562445	-0.018412
6	0.702569	-3.714763	0.004560

1	1.209971	-4.675168	0.024924
6	-0.702561	-3.714767	-0.003297
1	-1.209960	-4.675180	-0.023330
6	-1.479246	-2.562442	0.019281
1	-0.939358	-1.627400	0.074465
6	-2.865496	-2.465745	-0.004778
1	-3.443691	-3.385273	-0.025081
6	-3.567775	-1.248749	0.001714
1	-4.653265	-1.289137	0.020337
6	-2.958007	-0.000007	-0.020786
1	-1.878184	-0.000015	-0.074059
6	-3.567777	1.248742	0.001287
1	-4.653267	1.289134	0.019895
1	-3.443696	3.385257	-0.026240
6	-2.865501	2.465737	-0.005619
1	-0.939361	1.627422	0.073920

Table 77. $C_{18}H_{18}$ triplet (C_2h) optimized with UHF:

6	2.704232	-1.381276	0.226661
6	3.740672	-0.572057	-0.099696
1	4.626596	-0.980315	-0.559985
6	3.674595	0.903940	0.099699
1	4.520493	1.389729	0.559989
6	2.569990	1.617309	-0.226658
1	1.768128	1.073519	-0.692673
6	2.348256	3.013393	0.032132
1	3.198634	3.613292	0.310316
1	1.055073	4.684316	0.227974
6	1.091665	3.626814	0.022166
6	-0.131907	2.946323	-0.140824
1	-0.084644	1.889983	-0.315338
1	-1.469386	4.571352	0.227973
6	-1.411369	3.514808	0.022166
6	-2.608132	2.791586	0.032125
1	-3.508710	3.313113	0.310294
1	-1.856970	0.911308	-0.692650
6	-2.704231	1.381272	-0.226660
6	-3.740676	0.572058	0.099686
1	-4.626609	0.980322	0.559954
6	-3.674598	-0.903941	-0.099691
1	-4.520504	-1.389736	-0.559962
6	-2.569990	-1.617304	0.226658
1	-1.768122	-1.073509	0.692657
6	-2.348255	-3.013392	-0.032119
1	-3.198636	-3.613295	-0.310288
6	-1.091665	-3.626812	-0.022157
1	-1.055074	-4.684315	-0.227959

6	0.131911	-2.946322	0.140821
1	0.084653	-1.889981	0.315330
6	1.411370	-3.514810	-0.022174
1	1.469385	-4.571353	-0.227982
1	3.508709	-3.313112	-0.310317
6	2.608133	-2.791588	-0.032136
1	1.856978	-0.911318	0.692669

Table 78. $C_{18}H_{18}$ triplet (C_2h) optimized with UB3LYP:

6	-1.478203	-2.608565	0.131302
6	-0.718192	-3.722402	-0.036538
1	-1.204202	-4.674445	-0.237532
6	0.739137	-3.718302	0.036552
1	1.230495	-4.667592	0.237565
6	1.492876	-2.600213	-0.131310
1	0.955902	-1.697389	-0.399156
6	2.911616	-2.464725	0.016356
1	3.492470	-3.369748	0.169820
1	4.660780	-1.269044	0.140170
6	3.580501	-1.248856	0.017854
6	2.957013	0.008312	-0.073269
1	1.879970	0.005275	-0.172096
1	4.653554	1.295250	0.140182
6	3.573405	1.268977	0.017864
6	2.897687	2.481057	0.016369
1	3.473438	3.389335	0.169836
1	0.946312	1.702738	-0.399156
6	1.478206	2.608565	-0.131308
6	0.718193	3.722398	0.036547
1	1.204200	4.674438	0.237561
6	-0.739138	3.718299	-0.036549
1	-1.230493	4.667592	-0.237556
6	-1.492878	2.600210	0.131305
1	-0.955906	1.697384	0.399147
6	-2.911618	2.464726	-0.016361
1	-3.492471	3.369750	-0.169823
6	-3.580504	1.248858	-0.017858
1	-4.660784	1.269046	-0.140172
6	-2.957014	-0.008309	0.073264
1	-1.879971	-0.005267	0.172084
6	-3.573404	-1.268976	-0.017859
1	-4.653553	-1.295251	-0.140169
1	-3.473437	-3.389334	-0.169819
6	-2.897684	-2.481056	-0.016363
1	-0.946304	-1.702734	0.399128

Table 79. $C_{18}H_{18}$ triplet optimized with UCAM-B3LYP:

6	-2.713625	1.252990	0.160621
6	-3.749017	0.419170	-0.051350
1	-4.721311	0.825465	-0.317719
6	-3.627283	-1.036053	0.050997
1	-4.518748	-1.598315	0.316557
6	-2.467525	-1.686118	-0.160205
1	-1.629762	-1.082500	-0.488385
6	-2.211543	-3.089567	0.016116
1	-3.064806	-3.737306	0.190147
1	-0.875865	-4.721656	0.167722
6	-0.952179	-3.647500	0.022919
6	0.244406	-2.920683	-0.084727
1	0.154985	-1.850332	-0.202705
1	1.648136	-4.510691	0.167706
6	1.545027	-3.438786	0.022856
6	2.694295	-2.679439	0.015976
1	3.643278	-3.176468	0.190051
1	1.787176	-0.796984	-0.488597
6	2.713560	-1.252965	-0.160320
6	3.749135	-0.419163	0.051082
1	4.721645	-0.825479	0.316605
6	3.627321	1.036047	-0.051016
1	4.518797	1.598397	-0.316367
6	2.467536	1.686097	0.160229
1	1.629746	1.082476	0.488337
6	2.211557	3.089576	-0.016039
1	3.064791	3.737373	-0.189993
6	0.952158	3.647479	-0.022961
1	0.875828	4.721618	-0.167849
6	-0.244427	2.920635	0.084676
1	-0.155015	1.850280	0.202538
6	-1.545062	3.438787	-0.022874
1	-1.648146	4.510692	-0.167744
1	-3.643295	3.176477	-0.190159
6	-2.694332	2.679463	-0.015949
1	-1.787452	0.797127	0.489653

Table 80. $C_{18}H_{18}$ triplet optimized with UM06-2X:

6	-2.600676	-1.469273	-0.169230
6	-2.462402	-2.890453	0.015089
1	-3.367246	-3.463521	0.191007
6	-1.251212	-3.547590	0.022660
1	-1.256544	-4.625342	0.161661
6	0.000349	-2.912741	-0.075699
1	0.000216	-1.835807	-0.169722
6	1.252160	-3.547217	0.022696
1	1.257861	-4.624984	0.161574

1	3.368122	-3.462577	0.191188
6	2.463136	-2.889735	0.015265
6	2.601109	-1.468446	-0.168993
1	1.724851	-0.934522	-0.519818
1	4.638008	-1.221129	0.328931
6	3.708370	-0.729341	0.053694
6	3.708134	0.730389	-0.053829
1	4.637436	1.222468	-0.329688
1	1.724825	0.935231	0.520833
6	2.600778	1.469267	0.169395
6	2.462377	2.890355	-0.015379
1	3.367098	3.463440	-0.191832
6	1.251068	3.547492	-0.022916
1	1.256477	4.625170	-0.162495
6	-0.000369	2.912744	0.076080
1	-0.000240	1.835891	0.171050
6	-1.252261	3.547221	-0.022751
1	-1.257843	4.624917	-0.162158
6	-2.463166	2.889804	-0.015163
1	-3.368163	3.462591	-0.191231
6	-2.601135	1.468541	0.169289
1	-1.724915	0.934699	0.520326
6	-3.708243	0.729348	-0.053700
1	-4.637804	1.221154	-0.329181
1	-4.637509	-1.222445	0.328923
6	-3.708037	-0.730363	0.053618
1	-1.724512	-0.935234	-0.520124
